# Benchmarking Tree and Ancestral Sequence Inference for B Cell Receptor Sequences

**DOI:** 10.3389/fimmu.2018.02451

**Published:** 2018-10-31

**Authors:** Kristian Davidsen, Frederick A. Matsen

**Affiliations:** Computational Biology Program, Fred Hutchinson Cancer Research Center, Seattle, WA, United States

**Keywords:** ancestral sequence reconstruction, B cell receptor repertoire, phylogeny, benchmarking, antibodies

## Abstract

B cell receptor sequences evolve during affinity maturation according to a Darwinian process of mutation and selection. Phylogenetic tools are used extensively to reconstruct ancestral sequences and phylogenetic trees from affinity-matured sequences. In addition to using general-purpose phylogenetic methods, researchers have developed new tools to accommodate the special features of B cell sequence evolution. However, the performance of classical phylogenetic techniques in the presence of B cell-specific features is not well understood, nor how much the newer generation of B cell specific tools represent an improvement over classical methods. In this paper we benchmark the performance of classical phylogenetic and new B cell-specific tools when applied to B cell receptor sequences simulated from a forward-time model of B cell receptor affinity maturation toward a mature receptor. We show that the currently used tools vary substantially in terms of tree structure and ancestral sequence inference accuracy. Furthermore, we show that there are still large performance gains to be achieved by modeling the special mutation process of B cell receptors. These conclusions are further strengthened with real data using the rules of isotype switching to count possible violations within each inferred phylogeny.

## Introduction

B cells play a key role in adaptive immunity. After successful VDJ gene recombination of the variable part of the B cell receptor (BCR), and various selection steps, mature B cells are exported from the bone marrow. At this stage the mature B cells have not yet bound antigen and they are therefore referred to as naive. Upon infection some cells from this repertoire of naive BCRs will bind the infectious agent, initializing a cascade of events called affinity maturation leading to pathogen neutralization.

Affinity maturation is a micro-evolutionary process consisting of coupled mutation and selection. This essential process takes place in specialized anatomic compartments called germinal centers (GCs), with the objective of improving antigen binding of the BCR (1). Affinity maturation results in “clonal families” of thousands of B cells for each of the naive ancestors. Sequences in a family are related to a common naive B cell but with higher affinity BCRs and accumulation of mutations in their sequences.

The study of B cell evolution in the GCs is an important and active field of research including response to infections, mechanisms of vaccines (2) and immunological memory (3). Furthermore, the field has experienced a boost of interest and capability in recent years due to the advancements of high-throughput sequencing of BCR repertoires (Rep-Seq) (4). Rep-Seq now enables sequencing of BCRs on massive scale (millions of cells) and is being increasingly applied in different areas from vaccine studies (5, 6) to antibody engineering (7, 8). Following Rep-Seq, computational methods can be used to group the BCRs into clonal families, each consisting of the descendants of a single naive cell (9).

The events of the affinity maturation process can be interrogated by inferring the phylogenies of sequences within each such clonal family, as well as inferring ancestral sequences on the phylogenies. Phylogenetic methods have given great insight into the long and complex development process of broadly-neutralizing antibodies (10, 11). Phylogenetic methods are equally important for shorter-time-scale investigations of affinity maturation, such as of the response to vaccination (12). One may also use trees equipped with ancestral sequences to make statements about the strength of natural selection (13).

Given the importance of these methods to understanding affinity maturation, there has been surprisingly little validation of their performance in the parameter regime relevant to the study of affinity maturation. Although dozens of studies benchmarking phylogenetic methods via simulation in the general phylogenetic case have appeared since (14), methods for BCR sequences deserve special treatment because of special aspects of the evolutionary process of affinity maturation. These include:

1. The somatic hypermutation (SHM) process in affinity maturation is driven by purpose-built molecular machinery (15) that results in a highly context-dependent process with local sequence contexts that either favor (“hotspots”) or disfavor (“coldspots”) mutation (16, 17). The complexity of this process is at odds with both the usual phylogenetic assumption of independent and identical processes between sites and with the assumptions of commonly-used sequence simulators (18, 19) used for benchmarking.
2. Sampling and sequencing, especially for direct sequencing of GCs (20), is dense compared to divergence between sequences. Because the resulting sequences will have limited divergence between them, it raises the possibility that simpler methods with fewer free parameters such as parsimony would be an appropriate choice (21). Also, because of the resulting distribution of short branch lengths, zero-length branches and multifurcations representing simultaneous divergence are common. When these zero-length branches lead to a leaf, they represent a “sampled ancestor” – a sequence with an identical genotype to an ancestral cell. Because of these differences, previous conclusions about performance of phylogenetic estimators in the classical regime of millions of years of divergence need not hold here.
3. Rep-Seq typically sequences the coding sequence of antibodies, which are under very strong selective constraint in GCs. This contrasts strongly with the neutral evolution assumptions of most phylogenetic algorithms, as well as the neutral assumptions of the most common software used for phylogenetics benchmarks (18, 19).
4. In contrast to typical phylogenetic problems where the root sequence is unknown, one has significant information about the root sequence for BCR sequences. Even our current imperfect knowledge of germline genes greatly constrains the space of possible ancestral sequences compared to the typical phylogenetic case where the ancestor is completely unknown. Evolution of BCR sequences happens in a directed fashion from this ancestral sequence.

For these reasons, we believe that BCR-specific validation of phylogenetic tools is an essential prerequisite to their use.

Practitioners frequently use standard phylogenetic tools for BCR sequences. Many studies performing phylogenetic reconstruction on BCR sequences have used the PHYLIP package (22) such as the maximum likelihood (ML) tool dnaml (11, 23–25) or the maximum parsimony (MP) implementation dnapars (26–28). For general phylogenetics use, PHYLIP's dnaml is now less frequently used compared to faster or more feature-rich programs such as RAxML (29), PhyML (30), FastTree2 (31), and the most recent popular ML program, IQ-TREE (32). However, not all of these programs return ancestral sequence estimates so are less interesting for antibody researchers.

Four tools have been developed specifically for inferring BCR phylogenies: IgTree (33), ARPP (34), IgPhyML (35), and GCtree (36). IgTree aims to find the minimal sequence of events that could have led to the observed sequences (i.e., a maximum parsimony criterion), allowing a known root and sampled ancestors. ARPP is an implementation of a BCR specific ML model to infer ancestral sequences on trees produced by PHYLIP's dnaml. Both IgTree and ARPP have limited availability: IgTree is not available for download at all, while ARPP is only available for Windows. ARPP cannot be run from a script, thus we could not include it in this large-scale benchmark. IgPhyML adapts the Goldman-Yang (GY94) codon substitution model (37) by adding parameters to model the motif dependent mutation rate. However, to achieve a tractable likelihood the motif contribution is marginalized across codons to achieve a independent-across-codon likelihood function that works well with the usual ML setup. IgPhyML is built on codonPhyML (38) which is used for tree inference and likelihood calculations; ancestral sequence reconstruction can be done in a post processing step using an auxiliary script (provided in the supplement of (35)). GCtree ranks equally parsimonious trees found by PHYLIP's dnapars according to a likelihood function derived from a Galton-Watson branching process (39). In this branching process, the cellular abundance of a given genotype is used and therefore single cell data is a necessary requirement for optimal ranking with GCtree. Both IgPhyML and GCtree are freely available through GitHub. Additionally, we have implemented an alternative method, called SAMM v0.2, for ranking equally parsimonious trees based on the sum of log likelihoods of the observed mutations between nodes on a tree given a substitution model based on SHM motifs. This ranking is implemented using the SAMM package (40) and described in more detail in Methods.

To benchmark phylogenetic methods for BCRs, we desired a simulator for full-length BCR sequences that modeled context-sensitive mutation, natural selection on amino acids, and had publicly available source code. Many interesting simulators have different goals. Detailed mechanistic models have been proposed to model all cells and all interactions in a GC using first principles from biophysics (41–43). Others have suggested probabilistic frameworks modeling summary statistics of SHM (44, 45) and, as a middle ground between ultra fine grained models and plain summary statistics, models attempting to explain population level trends using systems of differential equations have been suggested (46). Even simulators that use a notion of sequence don't necessarily use nucleotides or model mutation in an accurate way. For example, (41) uses a reduced-size alphabet to obtain an appropriately rugged fitness landscape, while (47) use uniform per-site nucleotide mutation in the complementarity determining region and selection based on a subset of key residues.

No existing simulator fit our needs and so we designed a simple model of affinity maturation of BCR sequences in a clonal family. In this model, sequence fitness is solely a function of the amount of antigen bound by the BCR at equilibrium. Antigen binding is calculated using standard binding kinetics applied to a GC with B cells carrying BCRs with different sequences and affinities, competing to bind a limited amount of antigen. Our simple design is motivated by the observation that antigen binding is the main driver and limiting factor of affinity maturation (48). By modularizing the simulation code we have one module preforming mutation and proliferation as a neutral branching process and an optional module to change the birth/death rate through affinity selection.

This simulator has enabled a primary goal of our work: to benchmark methods for ancestral sequence reconstruction. Such methods infer sequences at ancestral nodes of a phylogenetic tree according to some optimality criterion. Ancestral sequence reconstruction is heavily used in BCR sequence analysis, in which it is common to synthesize and test ancestral sequences in order to understand the impact of historical substitutions on binding (49, 50).

A recent and independent effort by Yermanos et al. (51) did a benchmarking study using simulated BCR sequences without selection and compared phylogenetic method performance, including ML and MP tools. Our study has the following differences with this previous work:

- we simulate sequences under selection using an affinity-based model, which we show makes the inferential problem significantly more difficult,
- we compare accuracy of ancestral sequence inference,
- we include additional software tools, several of which are BCR-specific,
- we provide evidence that our simulations have similar characteristics to real data,
- and we use isotype data as a further non-simulation means of benchmarking methods.

This previous work also worked to understand the results of phylogenetic inference using a “toy” clonal family inference method with necessarily bad performance, whereas here we assume that clonal families have been properly inferred.

**Figure 1:**
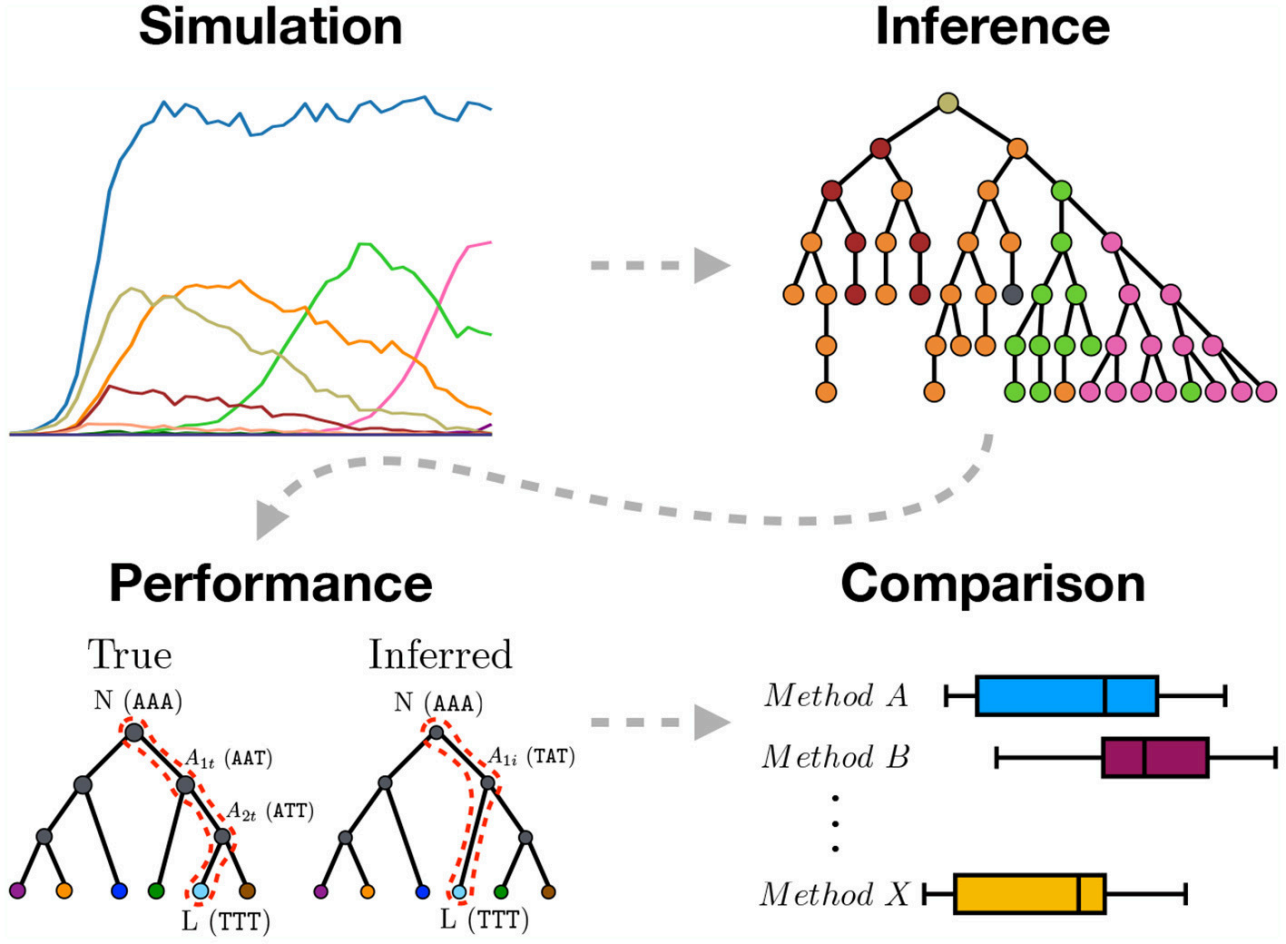
Graphical abstract summarizing the work presented in this paper. We use sequence simulation to establish a ground truth phylogeny from which a sample of sequences is used to infer the phylogeny using different inference methods. The inferred tree is then compared to the simulated true tree to measure inference performance. Lastly, the different inference methods are compared.

In this paper we attempt to answer some of the unresolved questions about BCR phylogenetic inference, including a benchmark of the performance of relevant phylogenetic tools (dnaml, dnapars, IgPhyML, IQ-TREE, GCtree and an undescribed SHM motif based tree ranking method), an investigation of the influence of SHM motifs; and a comparison between simulations with neutral or selection-based evolution (Figure 1). We apply our proposed sequence simulation framework to simulate under different realistic models that include SHM motifs and affinity selection. Finally, we show how the biological mechanism of isotype switching can be used to empirically test phylogenetic inference.

All simulation code is open source and can be found on our GitHub repository together with sequence data for the isotype validation (https://github.com/matsengrp/bcr-phylo-benchmark). All simulation data is organized to reproduce figures and is available for download on Zenodo (https://doi.org/10.5281/zenodo.1306301).

## Methods

Although statisticians have made substantial strides in proving identifiability (52, 53) of phylogenetic models and consistency (54) of inferential procedures, proving consistency of phylogenetic methods under context-sensitive BCR evolution models with selection is out of reach because no likelihood function is available. Therefore, we chose the general approach of simulating phylogenies, and benchmark tools based on their inference on samples from these known trees. As ancestral sequence reconstruction is of special interest among the users of BCR phylogenetics (11, 50, 55) we developed a metric to measure ancestral sequence reconstruction performance. In the following subsections we present these simulations and performance metrics, as well as a method to use empirical data to assess performance via the principle of irreversibility of isotype switching.

###  simulation

We devised two simulation strategies for BCR evolution: (1) a neutrally evolving branching process, and (2) a branching process with a birth/death rate controlled by BCR antigen binding. Both simulations start with a single naive sequence as a starting point for the tree simulation; this is evolved a number of generations to a population of BCR sequences from which a sample is drawn and used for inference. To get realistic starting sequences for the simulations we created a set of 288 naive sequences inferred by partis (56) from the healthy donor human single cell dataset in Briggs et al. (57). These sequences were selected because they have many unique unique molecular identifier (UMI) tagged reads, which gives a high confidence consensus over the full VDJ region. When a simulation run is initialized a naive sequence is drawn randomly from this set.

Our neutral model is controlled by two parameters which are used to control two Poisson distributions determining the simulation: the progeny distribution (λ) and the mutation generating distribution (λ_mut_). Each evolving sequence has its own λ which expresses the fitness of that sequence in comparison to the other sequences in the population (details below). All sequences have the same mutation probability i.e., λ_mut_ is the same for all sequences and constant throughout the simulation. The simulation starts with a single cell carrying the naive sequence; a draw from Pois(λ) will yield the number of progeny cells in the first generation. If a zero is drawn the cell dies, if one is drawn it propagates without division, if two is drawn it splits into two cells, etc. Next, for each progeny cell a draw from Pois(λ_mut_) will determine how many mutations to introduce into its sequence. Mutations are drawn either from a uniform distribution over both sites and substitutions, or using a context sensitive motif model (e.g., S5F (16)). Multiple mutations are introduced stepwise, one at a time, and if a context sensitive mutation model is chosen the sequence context is updated between each introduced mutation. The simulation process can be terminated in three ways: (1) when all cells have died, (2) at fixed time point *T*, or (3) when a fixed number of cells, *N*, has been reached.

As mentioned above, birth and death rates are controlled through the Poisson rate λ. One can think of this as measuring the level of T helper cell signal, in which lots of signal promotes proliferation while insufficient signal leads to death (1). In our neutral simulations, λ is held constant and is the same for all cells. For simulations with selection we use a very simplistic view of the maturation process, in which selection is purely driven by T helper cell signal which is strong for BCRs binding a lot of antigen and weak for BCRs binding little antigen. To translate this into selection in our simulation framework we devise a simple model to transform a BCR sequence into an affinity value, solve for its antigen binding and then use this to control λ, thus making it sequence dependent. In essence, this “affinity selection” is just a mapping between a BCR sequence and a λ; this enables us to use the same simulation framework for both neutral and affinity simulations. We emphasize that cells with a small λ will tend to draw a 0 from the Poisson distribution and die, so this framework incorporates cell death in addition to division and persistence.

Here we review the basics of fitness assignment; a detailed description of the model as well as model choices can be found in the Supplementary Material. For any BCR sequence indexed by *i*, its fitness is λ^(*i*)^ = *Y*(*x*), where *Y* is a transformation of some information, *x*, specified in the simulation. For a neutral simulation *Y*(*x*) is constant and independent of *x*, while for the affinity simulation *Y* is variable with respect to *x*. To model BCR sequence affinity we introduce the concept of a “mature sequence” which is the sequence with the highest attainable fitness in the simulation run. Once the simulation starts the mature sequence acts as an attractor to which evolution tends to converge by rewarding amino acid sequences closer to the attractor with higher λ. The choice of mature sequence is arbitrary so we chose to simulate it by randomly mutating the naive sequence until it accumulates a predefined number of amino acid substitutions. Next, the naive and mature sequence are assigned their own affinity values and the span between these define the affinity gain during affinity maturation. To calculate the affinity of a BCR sequence we calculate its amino acid Hamming distance to the mature sequence and transform this into an affinity value using an appropriate power function calibrated on the naive and mature sequences. We then model the BCR binding kinetics by defining a total GC volume with a constant concentration of antigen and solve for the B cells' antigen occupancy at equilibrium. Antigen occupancy is mapped to B cell fitness (λ^(*i*)^) using a logistic function returning a value between 0 and 2. These steps describe the general setup of calculating *Y*(*x*) for the affinity simulation.

**Figure 2:**
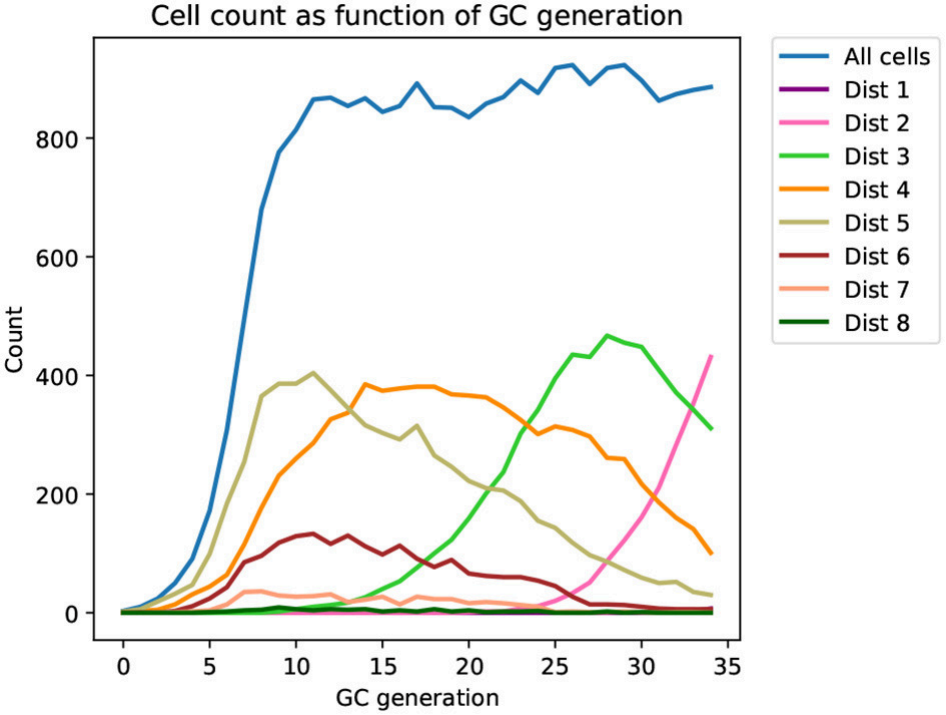
Time series of the distribution of cells at different distances from the mature sequence (Dist 1, 2, …, 8) as it appears in a typical affinity simulation (corresponding tree shown in Figure 3). The simulation is started from a single naive sequence, five amino acid substitutions away from the mature sequence (Dist 5), and simulated sequences converge toward the mature sequence as generations progress.

**Figure 3:**
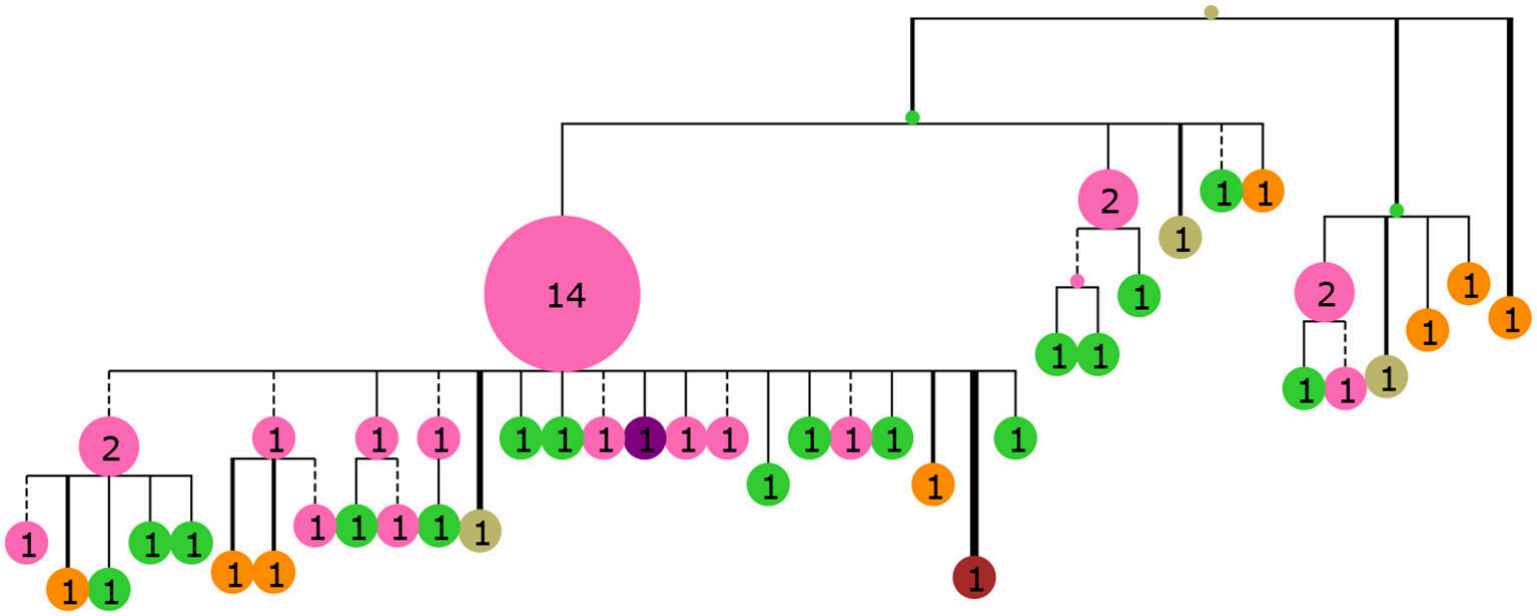
A collapsed tree made from 60 sequences sampled from GC generation 35 of the simulated population. Nodes are labeled with numbers indicating the number of collapsed tips (genotype abundance) and node size is proportional to this number. Branch lengths are Hamming distance between nucleotide sequences with dashed lines indicating purely synonymous mutations and solid lines indicating one or more non-synonymous mutations. Branch thickness is proportional to the number of non-synonymous mutations. The tree was rendered with ETE3 (58) and colored according to distance from the mature sequence with the same colors as Figure 2.

Inspection of the simulation runs confirm that affinity simulation recapitulate a number of desired properties (Figures 2, 3): (1) sequence evolution is converging toward the mature sequence, (2) cells are competing for the limited supply of antigen establishing a “carrying capacity,” and (3) favorable mutations are rapidly fixed through selective sweeps (59) analogous to clonal bursts (1, 20).

We set the expected number of mutations, introduced into the sequence at each mutation step, to be approximately 0.365. This corresponds to the frequently cited SHM rate at around 10^−3^ (60) given the average length of our naive BCR sequences of 365 nucleotides. We define λ_mut_ = 0.365 as the “normal” mutation rate, but because the estimates of SHM rate vary in the literature we also include half and double of this rate (λ_mut_∈{0.1825, 0.365, 0.73}) in all our simulations. We observe high correlation between the method performance across all three λ_mut_ (Figures S2, S3), showing that our conclusions are robust to differences in mutation rate. For neutral simulations the branching parameter (λ) and the population size termination criterion (*N*) are adjusted (λ = 1.5 and *N* = 75) to recapitulate summary statistics of the single cell GC experiment in Tas et al. (20) (Figure S25), following a similar procedure as DeWitt et al. (36). For the affinity simulations the branching parameter is cell-specific and adjusts dynamically, in the range between 0 and 2, according to antigen competition. Each affinity simulation uses 100 “mature” sequences, which act as a collection of targets for the convergent evolutionary process. These mature sequences are generated by randomly introducing 5 amino acid substitutions to the naive sequence (in depth description in Supplementary Material). Affinity simulations are run with an antigen concentration sufficient to maintain a cell population of approximately 1,000 cells, and after 35 generations a random sample of 60 cells is recovered for inference, again, roughly recapitulating summary statistics of the single cell GC experiment (Figure S26). We also performed intermediate sampling for the affinity simulation: in such cases 30 cells are sampled at generation 15, 30 and 45 and pooled to a total of 90 cells. Neutral simulations were run with 1,000 replicates and affinity simulations were run with 500.

### Inference methods

From each simulation run a subset of sequences was sampled and used for phylogenetic inference along with the correct naive sequence which was used as an outgroup. We tested a number of relevant tools either previously used in the context of BCR phylogenetic inference or with potential use in this field:

- dnaml v3.696: PHYLIP's implementation of ML using the F84 model (22)
- dnapars v3.696: PHYLIP's implementation of MP (22)
- GCtree v1.0: Branching process likelihood ranking of MP trees (36)
- SAMM v0.2: Mutation motif based likelihood ranking of MP trees (40)
- IgPhyML v0.99: GY94 codon model with hot/cold spot motif parameters (35)
- IQ-TREE v1.6.beta5 (IQT): Fast ML inference with many substitution models (32)

For all methods the naive sequence was used as an outgroup, furthermore, the naive sequence was used to reroot the tree after inference. For all methods no sequence partitioning was used. IQ-TREE was run using either JC, HKY or GTR nucleotide substitution models and using the “ASR” flag, but otherwise with default settings. IgPhyML was run as described in Hoehn et al. (35) and using the “-o tlr –motifs WRC_2:0,GYW_0:1,WA_1:2,TW_0:3,SYC_2:4,GRS_0:5 –hotness e,e,e,e,e,e” flags to optimize branch lengths and topology with NNI moves under the full HLP17 model containing a free parameter for all six degenerate hot/coldspots. dnaml was run using gamma distributed rates, a coefficient of variation of substitution rate among sites of 1.41, four rate categories and otherwise default parameters. dnapars was run using default settings. In the case of dnapars it is common to observe many equally parsimonious trees, and in those cases a random tree was drawn. GCtree was run as described in DeWitt et al. (36), passing both sequences and their abundances to the program. Both GCtree and SAMM use the equally parsimonious trees generated with dnapars for likelihood ranking, hence in the case when only a single MP tree is found, dnapars, GCtree and SAMM will by definition yield the same result.

The use of all the above methods has been described previously, except SAMM which is part of a statical framework to infer DNA mutation motifs using survival analysis (40). As it is well known that SHM is context sensitive (16, 17, 61) we ranked equally parsimonious trees according to their SHM motif likelihood, inspired by the branching process ranking of DeWitt et al. (36). Using SAMM we calculate the likelihood of the observed mutations given a tree equipped with ancestral sequences at the internal nodes (in this application from parsimony) and a motif model by using Chib's method (62) to integrate out event orders on the branches. This likelihood is then used to rank the equally-parsimonious trees, and the highest-ranked tree is chosen as the tree returned by SAMM. More detail on the likelihood calculation used in SAMM can be found elsewhere (40).

We would like to make it very clear that we use the same motif model for both simulating mutations and calculating SAMM likelihoods. This gives SAMM an unfair advantage, however, the selection process is not modeled as part of the motif model. We are not formally proposing SAMM ranking as a competing inference method, but rather as a yardstick with which to measure how much improvement would be possible taking a fully context-sensitive mutation process into account. On the other hand, SAMM has no inherent advantage on the isotype scoring experiment, and it is limited to the MP trees.

### Genotype collapsing

Due to our focus on ancestral sequence inference we have adopted the use of genotype collapsed trees from DeWitt et al. (36) throughout this work. Briefly, a genotype collapsed tree is made by inferring a phylogenetic tree, inferring ancestral sequences at the internal nodes and recalculating the branch lengths as Hamming distances between the node sequences. In the branch length recalculation step nodes are “collapsed” if their sequences are identical, thereby collapsing tips upwards and adding observations to internal nodes (Figure 3). Genotype collapsing deals conveniently with the very short branch lengths, typically observed in binary trees for BCR sequences, since these most often collapse into a single node.

### Tree and sequence reconstruction metrics

We scored trees both in terms of tree structure and in terms of ancestral sequence inference. For tree structure, we used the commonly used Robinson-Foulds (RF) distance (63), which is half the size of the symmetric difference between the sets of bipartitions obtained by cutting each edge. We define bipartitions using both tips and sampled internal nodes, as opposed to standard RF using only tips. Because we perform RF on genotype-collapsed trees, this measure in fact combines accuracy estimation of ancestral sequences and tree topology.

We also used several means to more directly compare ancestral sequence reconstructions: the “most recent common ancestor” (MRCA) metric, and the “correctness of ancestral reconstruction” (COAR) metric. The MRCA metric compares ancestral sequences on the true vs. the inferred phylogeny in a way that does not depend on agreement between the two topologies. Specifically, the MRCA distance is calculated by iterating through all pairs of leaves. For each such pair there is a well defined MRCA node on the tree. The MRCA metric is the average Hamming distance between the inferred and the true ancestral sequence for these pairs. Using *i* and *j* (*i*≠*j*) to iterate over all combinations of pairs of leaves to find their true (*T*_*i, j*_) and inferred (*I*_*i, j*_) most recent common ancestor, this can be written as:

$$\begin{array}{l} \overset{N}{\underset{i=1}{\sum }}\overset{N}{\underset{j=i+1}{\sum }}d_{H}\left(T_{i,j},I_{i,j}\right)/\left(N\left(N-1\right)/2\right)L. \end{array}$$

Here *N* is the number of leaves and *L* is the length of the sequence. Thus, MRCA gives an overall view of how ancestral sequence reconstruction is performing.

There is also a special interest in benchmarking tools to reconstruct a lineage of ancestral sequences going from the root (the naive sequence) to a tip of interest (11, 55). Hence, we developed the COAR metric which is measuring the average number of sequence mismatches across all true vs. inferred lineages going from the root to any tip. It is not initially obvious how to compute such a distance if the true and inferred lineage contains a different number of nodes. We solve this problem by finding the node to node comparison that minimizes the distance while maintaining the root-to-tip order. Please see the Supplementary Information for details on COAR metric calculation.

We chose COAR as our principal metric for comparison because it was well correlated with other metrics (see section Results) and because it reflects how researchers use ancestral sequence reconstruction of BCRs.

### Isotype scoring

We used sequences with isotype information as another means of characterizing phylogenetic accuracy. The isotype-determining constant region is located downstream of the heavy chain BCR variable region, and isotype changes through a process called class-switch recombination. In mice the isotype constant regions are ordered, from closest to furthest to the J gene: IgM, IgG, IgE, then IgA. Naive BCRs use IgM, but during affinity maturation isotype switching can occur by looping out one or more of the constant regions. For instance if IgM is looped out the resulting BCR is IgG and if IgM, IgG, and IgE is looped out the resulting BCR is IgA. Because the isotype is physically removed from the chromosome this process is irreversible, hence a parent cell with an IgA BCR can never give rise to a child cell of IgM isotype.

We use the irreversible nature of isotype switching to measure the performance of tree inference by mapping back isotype labels to the nodes on the inferred tree and counting the number of nodes with an edge to a child that violate the rules of isotype switching. We use the BCR data from Laustsen et al. (64) which is generated with unique molecular identifier (UMI) technology and primers targeting the isotype region on splenocyte whole mRNA from five outbred mice undergoing an immunization campaign. After extensive quality filtering using pRESTO (65) we ran partis (9) to partition sequences into clonal families. These clonal families were filtered based on having minimum 10 and maximum 200 unique sequences and containing at least two different isotypes. Furthermore, we discarded all clonal families where inference exceeded 24 h of compute time for any single tool on a single core. This left 697 clonal families to do isotype validation.

We defined an isotype mismatch as an observed violation of the isotype switching order (namely the order IgM, IgG, IgE, IgA). That is, an edge connecting a parent and a child node is an isotype mismatch if the isotype order of the parent is farther along the order than its child (Figure S18). To calculate the “isotype score” we iterate over all the tips and use each tip as a starting point to collect the list of isotypes between this tip and the root. This list is made by progressing from a tip to the root and collecting isotypes sequentially, however, unobserved internal nodes will not have an associated isotype and therefore they “reverse inherit” the isotype from their child. Once this list has been filled, each edge is evaluated and if an isotype mismatch is encountered the parent node is marked as a violator. The number of isotype switching violations is found by counting all the violator nodes.

This sum is dependent upon the shape of the inferred tree, potentially leading to a bias associated with each inference tool. To address this, for each inferred tree we created 10,000 samples of trees with the same topology but shuffled labels and from these we calculated a “baseline” isotype score to be expected given this topology. We divided the violation count by the baseline to obtain the final isotype score.

### Comparison to joint reconstruction

There are two approaches to maximum-likelihood ancestral sequence reconstruction. For joint reconstruction, one infers the collection of ancestral sequences that jointly maximize the likelihood of the sequence data given the tree and a substitution model (66). For marginal reconstruction, one infers the maximum likelihood ancestral sequences at each internal node individually, marginalizing over all the possible states of the other internal nodes. Under the maximum parsimony objective, ancestral sequence reconstruction is an inherent part of the tree construction and thus it is conceptually more similar to a joint ancestral sequence reconstruction.

All the ML based tools (dnaml, IgPhyML, and IQ-TREE) we test use marginal reconstruction, raising the question of whether this could influence the results of our benchmark and if the relatively good performance of parsimony could be explained by it being a joint-reconstruction technique. In order to investigate this question, we applied the FastML tool (66), capable of doing both joint and marginal ancestral sequence reconstruction. FastML was run using the HKY model and neighbor joining to build trees resulting in two reconstructions with the same tree: one joint and one marginal reconstruction. One thousand simulations under neutral and affinity simulation was performed using the previously defined three mutation rates. Finally, the joint and marginal reconstructions were compared with IQ-TREE as a visual reference (Figures S13–S17).

### Boxplot layout

Tool performance is plotted in boxplots. Colored boxes cover from lower to upper quartiles, with the median marked by gray vertical lines and whiskers extending to 1.5 times the interquartile range. Points beyond the range of the whiskers (outliers) are hidden for clarity. Red triangles mark the mean metric value of all simulations, with 1,000 replicates for neutral and 500 replicates for affinity simulations, with an overlapping horizontal red line showing the 95% confidence interval of the mean. Confidence intervals on the mean were computed using non-parametric bootstrapping, using sampling with replacement on the set of metric values to generate 10,000 bootstrap replicates (67). Tools are ordered according to their mean metric values.

## Results

### Metrics Are correlated

**Figure 4:**
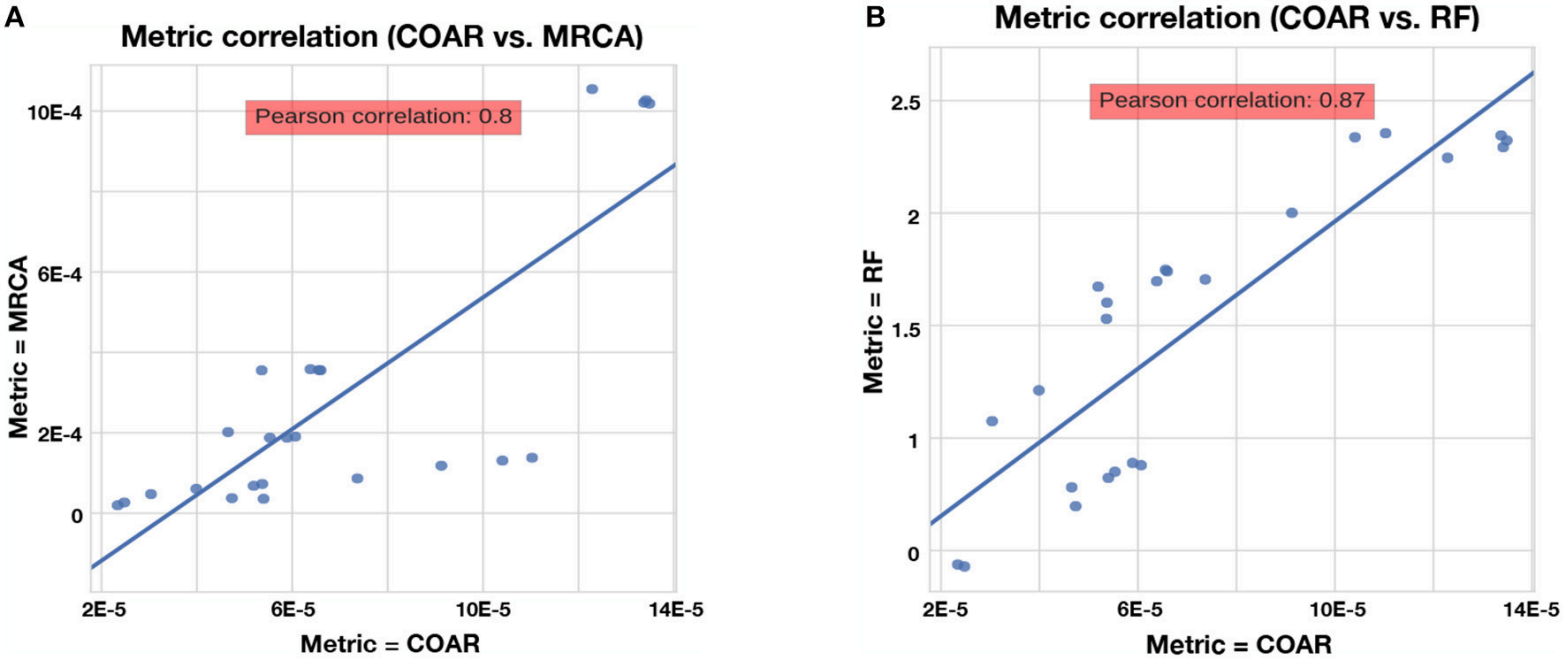
Correlation between metrics for the neutral simulation across the three mutation rates described in section Results. Same trend is true for affinity simulation (Figure S1). **(A)** Correlation between COAR and MRCA metrics. **(B)** Correlation between COAR and RF metrics.

The RF, MRCA, and COAR metrics are highly correlated, with COAR being the most central metric (Figure 4). We checked this for both neutral and affinity simulation and over a range of mutation parameters (Figure S1) and conclude that the high correlation between metrics is robust over many parameter choices. To reduce the number of comparisons we chose COAR as our principal metric because this was the most central metric as well as being interpretable as the expected number of per-site errors per reconstructed lineage. However, all metrics have been run on all simulations (see Supplementary Figures), except RF distance which does not deal well with reoccurring sequences that appear multiple times in the affinity simulation.

### Joint and marginal reconstruction performs equally well

We found that joint reconstruction does not have an advantage over equivalent methods using marginal reconstruction according to our criteria. To investigate this question, we ran default FastML v3.1 (66) with neighbor-joining tree inference to infer ancestral sequences with both joint and marginal reconstruction over a range of simulation methods and parameters. Using our three performance metrics: RF, MRCA and COAR, the two reconstruction methods performed essentially identically (Figures S13–S17). Because none of the ML methods initially tested had available joint reconstruction implementations, we cannot make specific conclusions about their performance using joint reconstruction. However, the fact that between joint and marginal reconstruction perform essentially identically is suggestive that this may be a general phenomenon in this parameter regime.

### Methods differ in performance consistently across simulations

**Figure 5:**
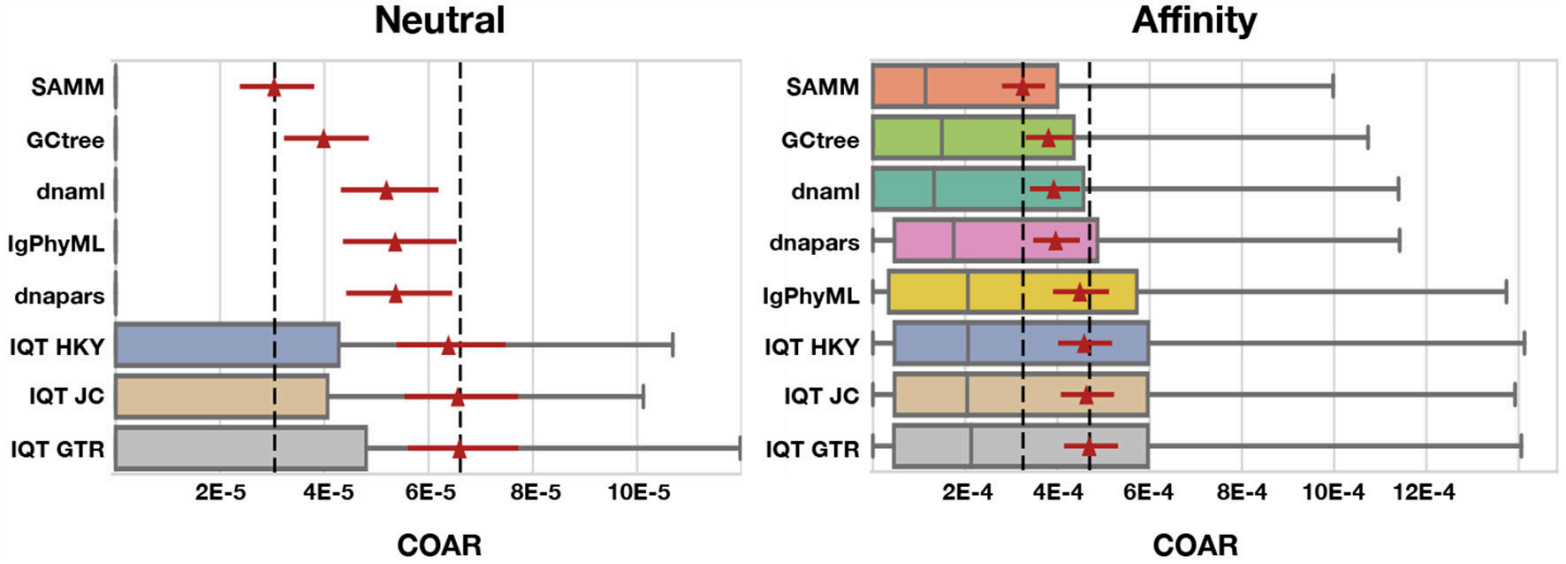
COAR performance for different tools under neutral and affinity simulation using normal SHM rate (λ_mut_ = 0.365) and mutations drawn from the S5F motif model. Colored boxes cover the lower to the upper quartiles, with the median marked by gray vertical lines and whiskers extending to 1.5 times the interquartile range. Points beyond the whiskers (outliers) are hidden for clarity. Red triangles mark the mean COAR value of all simulations (1,000 replicates for neutral and 500 replicates for affinity simulations) with the overlying red lines showing the 95% confidence interval found by bootstrapping the mean with 10,000 replicates. Black dashed lines mark highest and lowest mean COAR values. Tools are ordered according to their mean COAR value.

We observe similar trends across varying simulation methods, performance metrics, and mutation rates. A higher mutation burden (λ_mut_) leads to more complex trees resulting in decreased inference performance, and this is true for all methods and performance metrics (Figures S4–S10). Tools perform better on neutral simulation compared to affinity simulations (Figure 5), which is to be expected due to the added complexity of the affinity simulation. Overall, the distributions of performance metrics are heavy tailed with several outliers far outside of the interquartile range. We have chosen to hide such outliers for the interpretability of our boxplots but their impact can be observed in the means (red triangles) and their confidence intervals.

We find that SAMM and GCtree, which rank equally-parsimonious trees, perform better than a uniformly-selected equally parsimonious tree from dnapars. For all 15 tests across mutation rates, performance metrics and simulation methods SAMM is better than dnapars while GCtree is better than dnapars 13/15 times (Figures S4–S10). SAMM is the best ranked tool 12/15 times and often with a substantial margin to the second best. Thus the equally-parsimonious tree set contains better and worse trees, and the likelihood ranking of these is effective at distinguishing between them. However, given that SAMM were using the S5F model for likelihood calculations on simulated mutations also drawn from an S5F motif model, it should be not surprise to see that SAMM consistently outperforms all other tools.

Because SAMM is constrained by dnapars and the criterion of only ranking equally parsimonious trees, we consider the performance of SAMM compared to other tools as a conservative estimate of the potential improvement available when correctly modeling SHM motif bias. As a control, we note that when mutations are drawn from a uniform distribution over sites and substitutions, SAMM is not any better than dnapars (Figures S11, S12) showing that SAMM's performance can be ascribed to the mutational context bias. Thus, we can use the performance difference between SAMM and dnapars to measure how much inference performance can improve by incorporating SHM motif bias.

Simulated datasets include information on sequence abundance, which enables good performance of the GCtree method. Normally, phylogenetic trees are made from a set of unique sequences while the cellular abundance of each sequence, referred to as genotype abundance, is discarded. GCtree, on the other hand, utilizes this genotype abundance information by ranking equally parsimonious trees via a likelihood using abundances. Our results show that GCtree is the second best performing tool, and consistently better than picking a random equally parsimonious tree, indicating that the integration of genotype abundance information does improve tree inference. Here GCtree is given the correct abundances, giving an upper bound on the performance gain obtainable by incorporating abundance information. In a situation with real data GCtree would rely on single cell data to gain estimates of genotype abundances; while single cell data is becoming more widespread (57, 68–70) the majority of Rep-Seq studies are still based on bulk RNA sequencing resulting in unknown genotype abundances.

Performing third best after SAMM and GCtree comes dnaml and dnapars, both with similar performance, after that IgPhyML and lastly the three mutation models implemented in IQ-TREE which are all performing very similarly (Figure 5). dnapars performs slightly better than dnaml in neutral simulations while the opposite is true in affinity simulations. Practically, the difference between the two programs is so small that we suggest users to choose whichever program they find to be fastest or most convenient to use for their application.

Surprisingly, on simulated sequences IgPhyML performs consistently worse than the simpler dnaml or dnapars alternatives. Although, it is clear from the SAMM results that SHM motifs are present and provide useful information for inference, it does not seem to improve IgPhyML performance beyond SHM naive methods such as MP. IgPhyML's model was preferred (by likelihood ratio test) in the examples provided in the paper introducing it, which were large trees of long-term broadly-neutralizing anti-HIV antibodies (35). We suspect that IgPhyML's model is too rich for the less complex data provided here.

All three IQ-TREE methods, using different mutation models, perform consistently worse than any other tool tested in this study. We find it surprising that IQ-TREE using the HKY model is so far off dnaml using F84 despite the high similarity between the two substitution models. We therefore conclude that implementation differences e.g., tree space search, convergence criteria etc. must be the reason for this discrepancy, which is in concordance with our observation that IQ-TREE is much faster than dnaml.

### Isotype data confirms that raw parsimony can be improved by likelihood ranking

The results of our investigation using isotype were somewhat inconclusive. This measure had an extraordinarily large variance observed in both the confidence intervals and the changed rankings upon rerunning the analysis (Figure S19). Although SAMM did perform best among all tools when using a custom motif model fitted on the whole isotype dataset (using means for ranking), the difference to other tools was small relative to the variance, thus we cannot conclude from this comparison that SAMM is better than the next few tools.

**Figure 6:**
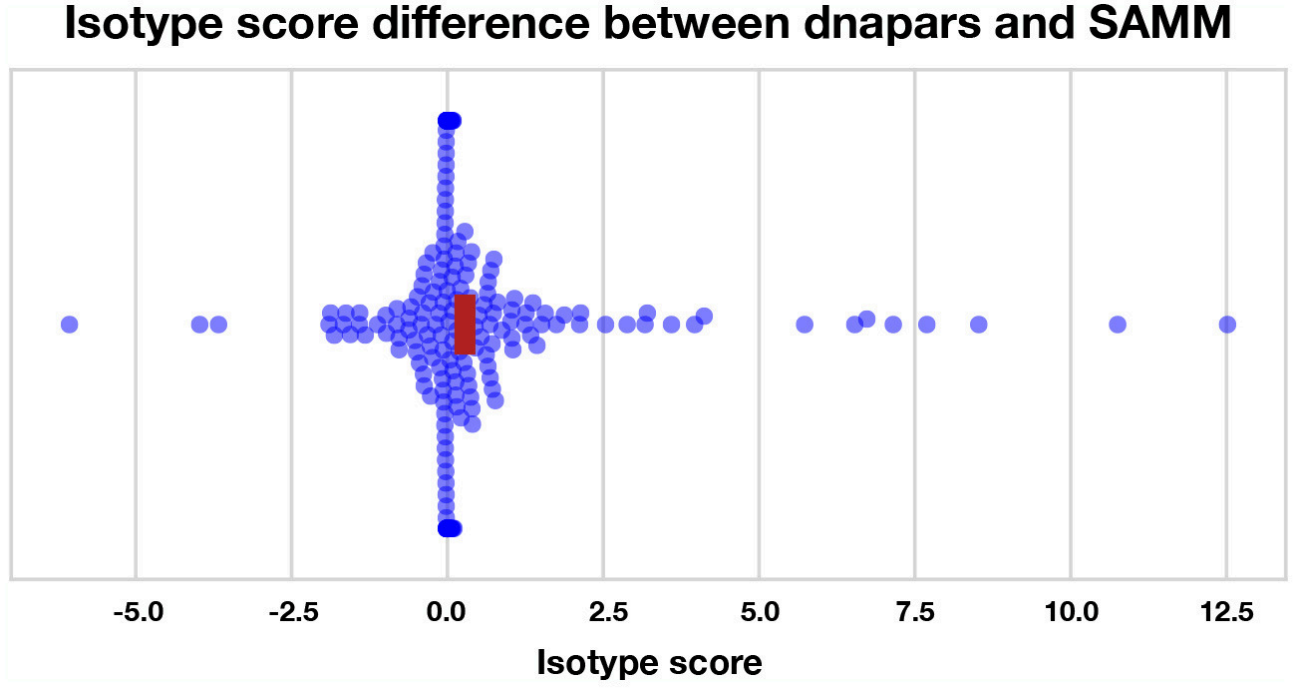
Isotype score differences between dnapars and SAMM for all clonal families with non-zero isotype score. Positive points are clonal families where dnapars had higher (worse) isotype score than SAMM and vice versa for negative values. The horizontal width of the red rectangle marks the 95% confidence interval of the mean difference.

We find that most methods are slightly, but significantly, better than dnapars (Figure S19). Furthermore, we find that SAMM improves upon raw parsimony (Figure 6), again confirming the notion that the SHM mutation process is important and contains residual information not captured by the parsimony objective. Notably, the parsimony ranking of GCtree is also significantly better than dnapars (Figure S19) despite the fact that this dataset did not contain genotype abundance information. This indicates that the branching process prior used by GCtree can also yield useful results using the tree topology alone. Testing the full potential of GCtree would require a single cell dataset and this may also result in even better performance. However, we emphasize that the difference in the isotype score distribution between dnapars and the other methods is quite small, especially when compared to the variance. Indeed, there are many trees for which dnapars performed much better than SAMM according to this metric (Figure S19, points < 0).

## Discussion

In this work we have benchmarked the performance of phylogenetic algorithms for use in B cell sequence analysis, with a special emphasis on ancestral sequence reconstruction. Our sequence simulation deviates from the standard independent-across-nucleotides models, often used in such benchmarking, by both introducing mutations using a realistic SHM motif model and rewarding convergent mutations via an affinity model of the binding equilibrium between BCRs and antigen. To our knowledge this is the first simulation method to model affinity maturation using BCRs represented as DNA sequences such that selection is based on the corresponding amino acid sequences. Inference based on affinity simulated sequences is more challenging, resulting in ~10 fold higher COAR values (Figure 5), underlining the importance of considering selection to get realistic error estimates on BCR phylogenetic reconstruction. Still, the average COAR values for affinity simulation is 0.0003–0.0005 which translates to an expectation of 1–2 total nucleotide errors in a lineage with 5 heavy+light chain BCR sequences reconstructed (~3,600 nucleotides). With the added benefit that about 1/3 of these expected mutations will be silent, reconstruction of BCR affinity matured lineages using ancestral sequence reconstruction in this parameter regime appears to be of high fidelity. However, this estimate should be tempered with the fact that the correct naive sequence was provided to the algorithm, and the general fact that complex processes happening in real data can make the problem significantly harder. In real applications there will be uncertainty in the inference of the naive sequence. In cases where an erroneous naive sequence is used in tree reconstruction, such nucleotide errors are likely to propagate toward the tips of the tree, increasing the expected number of errors.

Our simulations generally follow same summary statistics as a single instance of germinal center maturation starting from an unmutated naive B cell (Figures S25, S26). However, upon repeated exposures, germinal center maturation is more likely to be based on memory recall e.g., chronic or seasonal infections like HIV and influenza (71). Memory recall will naturally accumulate more mutations than maturation on a naive B cell and hence will constitute a more complex reconstruction task. As we do not simulate the conditions of memory recall our results cannot be directly applied to such cases, however, we do expect that in such cases the success of reconstruction is lower and that the expected number of nucleotide errors in a reconstruction is substantially higher than the expectations reported above. It also follows from the simulation summary statistics (Figures S25, S26) that our simulated trees are quite densely sampled, giving rise to sampled ancestors and short branch lengths. This stands in contrast to typical repertoire-wide data where clonal families are sampled more sparsely and therefore have longer branches on their corresponding phylogenetic trees. The short branch lengths of our simulations may favor simpler reconstruction methods such as parsimony. Because of these limitations our findings are not directly applicable to repertoire-wide datasets, although they do indicate that we cannot assume the results of simulations in the classical long-branch phylogenetic regime (e.g., (14)) hold for all cases of B cell lineage evolution.

Looking at the more subtle differences between tools two observations stand out: first, accounting for SHM motifs is the biggest contributor to accuracy, and second, implementation matters. The performance of SAMM on simulations clearly shows how SHM motifs leave a useful trace that can be integrated into an inference method. One such method is the HLP17 model used by IgPhyML (35), but it may suffer from noisy parameter estimates in cases with relatively few sequences per clonal family. An extension to IgPhyML may alleviate these problems by either fixing the hot/cold spot parameters with a predetermined motif model, or the means of combining information across clonal families. Yet, there are still reasons to attempt other ways of integrating SHM motifs, as well as other affinity maturation specific information like genotype abundances, into inference methods in more principled ways than mean field approximations or likelihood ranking of MP trees. Our benchmark also gives a reminder that implementation matters. Under otherwise similar substitution models two different implementations (dnaml and IQ-TREE) vary substantially and consistently in performance. We do not know what causes these differences, but we speculate that tree space sampling could be a critical point as this appears to be the most important difference between these two implementations, and because IQ-TREE experiences the same pathologies with multiple different substitution models. IQ-TREE's heuristics were probably tuned with the traditional phylogenetic case (of deeply diverging sequences) in mind, which is different from our use case.

BCR isotype switching is an irreversible event and contains useful information about the phylogenetic relationship among BCR sequences in the same clonal family. We observed that the two MP tree ranking methods (SAMM and GCtree) did significantly decrease the isotype score compared to picking a random equally parsimonious tree, thus confirming our simulations. Despite this it appears to be very difficult to use the isotype score as an empirical performance metric because of its high variance. We believe that this is in part due to sparse sampling of the clonal families (only few tens of sequences out of the thousands evolved in a GC). In such cases, incomplete sampling can cause penalization of correct reconstructions because of missing observations and the isotype score will not reach zero even with perfect reconstruction. However, on average the best reconstructions should have lower isotype scores than the worst reconstructions. With better sampling and more clonal families we expect the isotype score to be better resolved, with lower variance, and then it may be a more useful metric for assessing the performance of BCR phylogenetic inference, or simply used as a constraint in the inference model itself (72).

In this work we provided phylogenetic algorithms with the correct naive sequence. The impact of naive sequence uncertainty was in a way benchmarked by Yermanos et al. (51), in which they used a coarse method for clonal family inference and then asked if phylogenetic methods could later disentangle the families. Both our study and Yermanos et al. (51) leave open the question of the performance of phylogenetic methods when supplied with a potentially noisy estimate of the naive sequence supplied by current clonal family inference tools. We will perform the appropriate benchmarking as part of our future development of methods to perform phylogenetic reconstruction and naive sequence estimation simultaneously.

In this work we also have not tested the impact of insertion-deletion (indel) mutations, which do happen in BCR phylogenies (61, 73, 74). Current tools leave a lot to be desired for ancestral sequence inference in the presence of indels, as in our experience they “fill in” nucleotides at every site of an ancestral sequence inference, even if a gap is clearly the right choice. In addition, indels are not treated as the informative characters they are in mainstream phylogenetics software; rather, they are treated as missing data. Benchmarking phylogenetic tools would also require benchmarking the alignment step, which has an effect on ancestral sequence reconstruction accuracy (75). Nevertheless, this will be another important focus for future tool development and ancestral sequence reconstruction benchmarking within the field of BCR phylogenetic reconstruction.

## Author contributions

KD carried out the data analysis, otherwise KD and FM equally contributed to this work.

### Conflict of interest statement

The authors declare that the research was conducted in the absence of any commercial or financial relationships that could be construed as a potential conflict of interest.
