# Supplementary material for: Benchmarking Tree and Ancestral Sequence Inference for B Cell Receptor Sequences
Source: Front Immunol. 2018 Oct 31;9:2451. doi: 10.3389/fimmu.2018.02451 (PMC6220437; doi:10.3389/fimmu.2018.02451)
Supplement: Supplementary file 1 [file Image_1.pdf]

Benchmarking tree and ancestral sequence inference for B cell receptor sequences

Kristian Davidsen & Frederick A. Matsen IV†

Fred Hutchinson Cancer Research Center
†corresponding author: matsen@fredhutch.org

Supplementary Materials

Metrics are correlated in affinity simulations

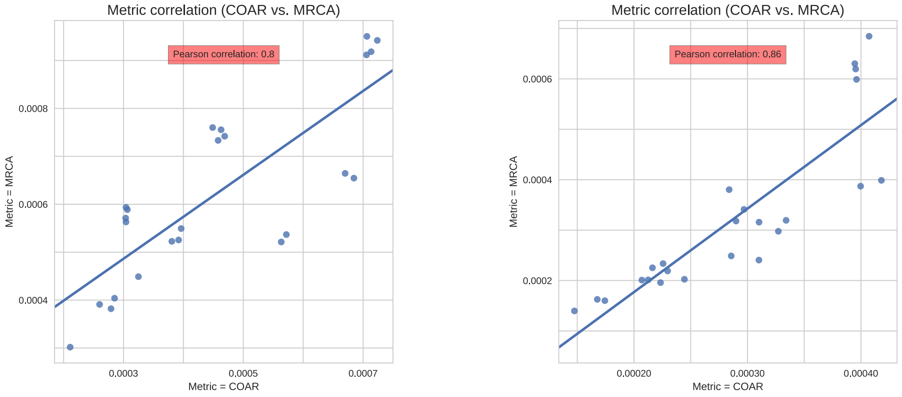

(a) (b)

Figure 1: Metric correlations for affinity simulations across three different mutations rates ($\forall \lambda_{\text{mut}} \in \{0.1825, 0.365, 0.73\}$). a) Single sample. b) Three samples, with two intermediate sampling times.

Metrics are robust across different mutation rates

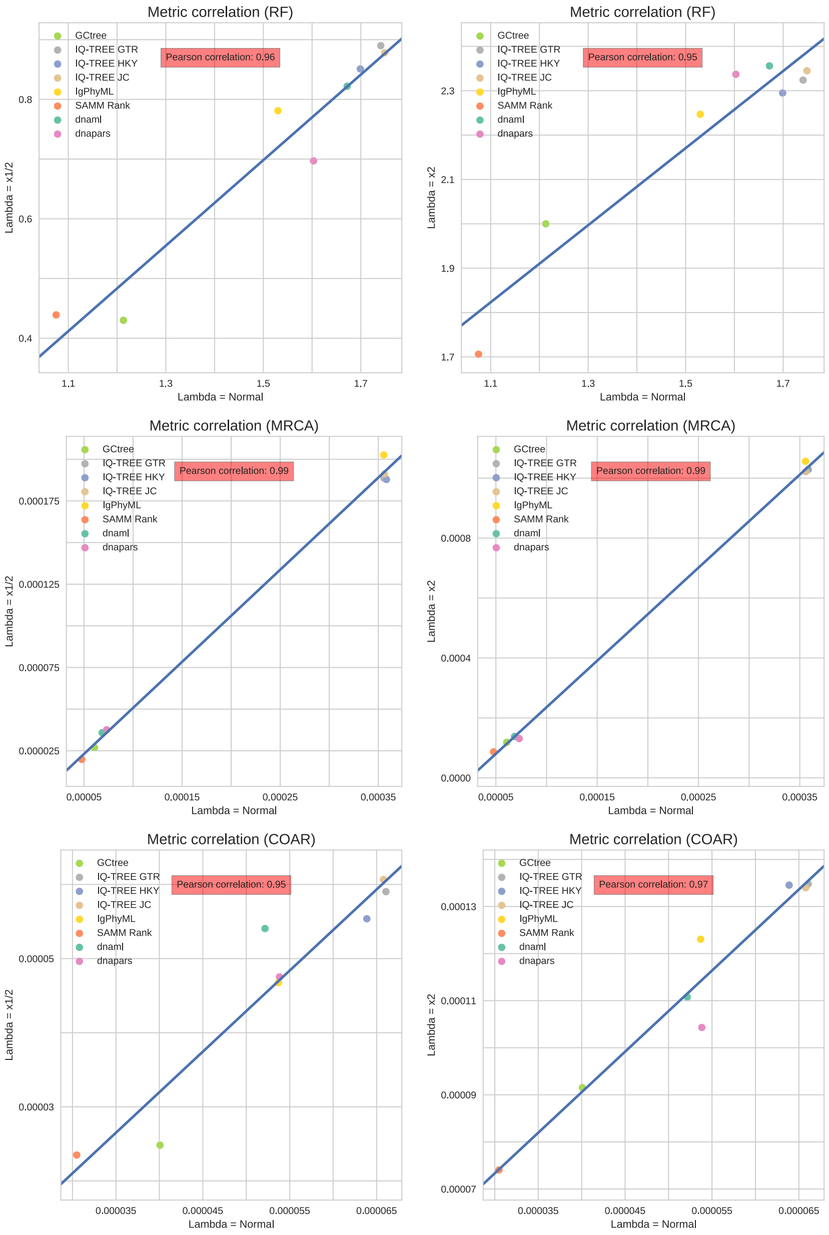

Figure 2: Correlation between the average performance of the methods tested at different mutation rates for neutral simulations over all three performance metrics.

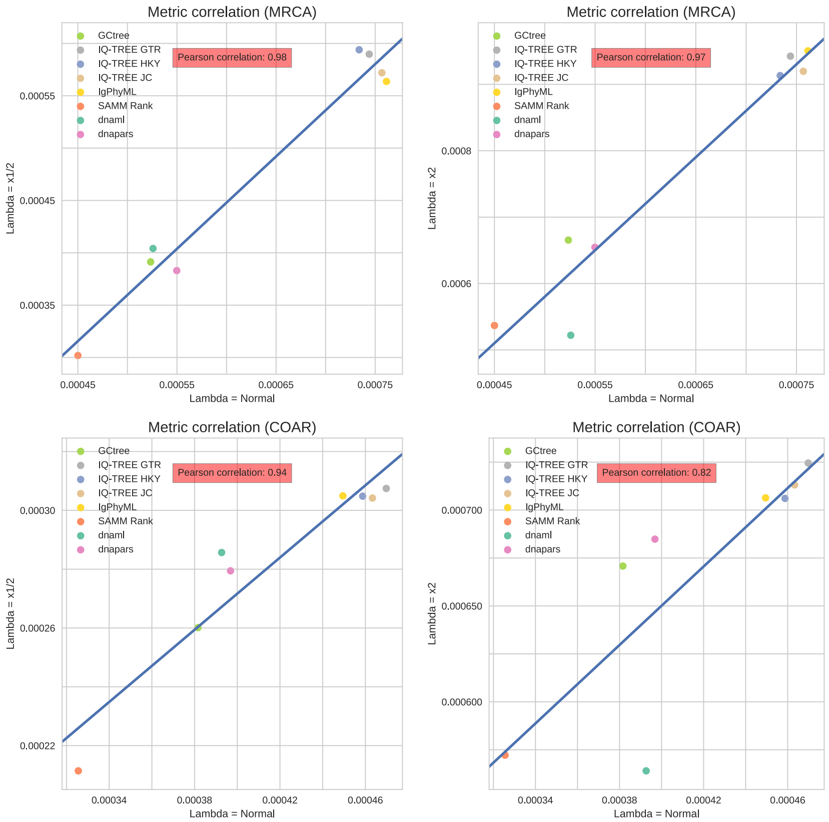

Figure 3: Correlation between the average performance of the methods tested at different mutation rates for affinity simulations over the two performance metrics (RF distance excluded because of recurring sequences in the simulated phylogeny).

Benchmarking results using COAR

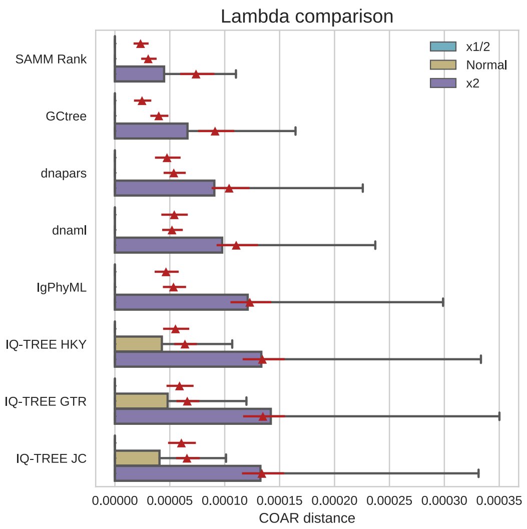

Figure 4: Neutral simulation showing COAR metric for mutation rates: “x1/2” = 0.1825, “Normal” = 0.365, and “x2” = 0.73.

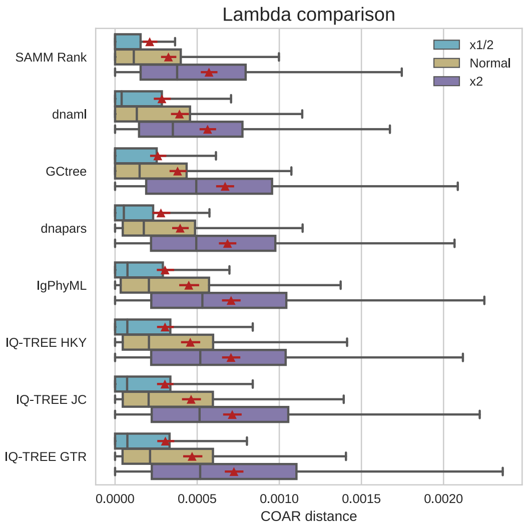

Figure 5: Affinity simulation showing COAR metric for mutation rates: “x1/2” = 0.1825, “Normal” = 0.365, and “x2” = 0.73.

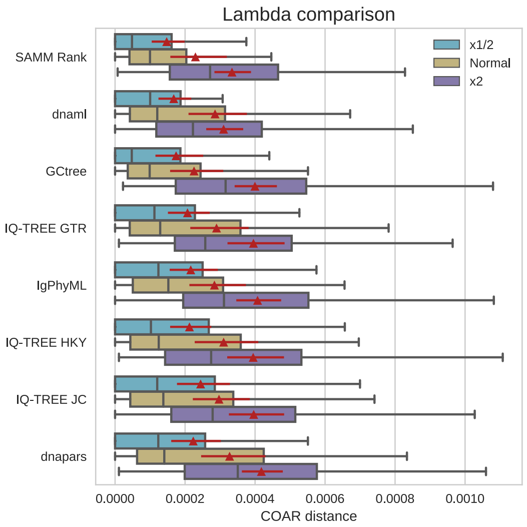

Figure 6: Affinity simulation with intermediate sampling (GC generation 15, 30 and 45) showing COAR metric for mutation rates: "x1/2" = 0.1825, "Normal" = 0.365, and "x2" = 0.73.

Benchmarking results using MRCA

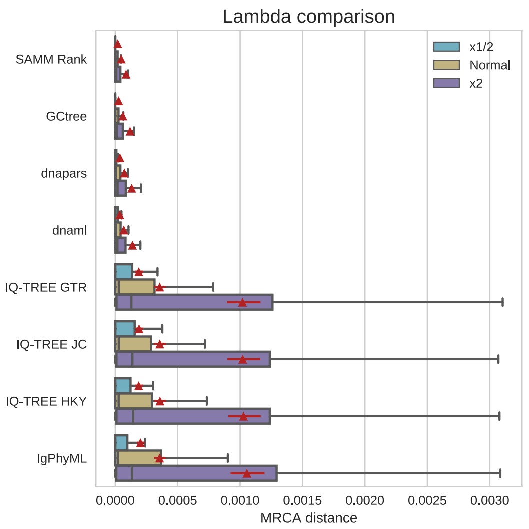

Figure 7: Neutral simulation showing MRCA metric for mutation rates: “x1/2” = 0.1825, “Normal” = 0.365, and “x2” = 0.73.

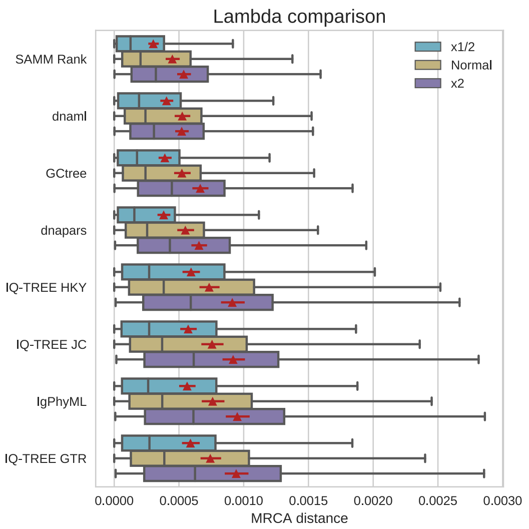

Figure 8: Affinity simulation showing MRCA metric for mutation rates: “x1/2” = 0.1825, “Normal” = 0.365, and “x2” = 0.73.

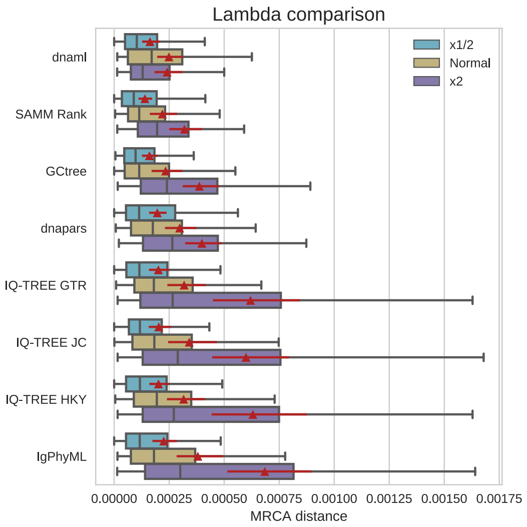

Figure 9: Affinity simulation with intermediate sampling (GC generation 15, 30 and 45) showing MRCA metric for mutation rates: "x1/2" = 0.1825, "Normal" = 0.365, and "x2" = 0.73.

Benchmarking results using RF

For affinity simulation, RF distance plots are excluded because of recurring leaf sequences in the simulated phylogenies.

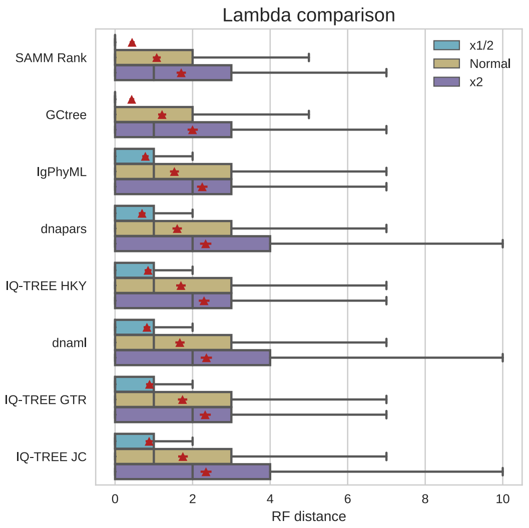

Figure 10: Neutral simulation showing RF metric for mutation rates: “x1/2” = 0.1825, “Normal” = 0.365, and “x2” = 0.73.

Benchmarking results using COAR and uniform mutation model

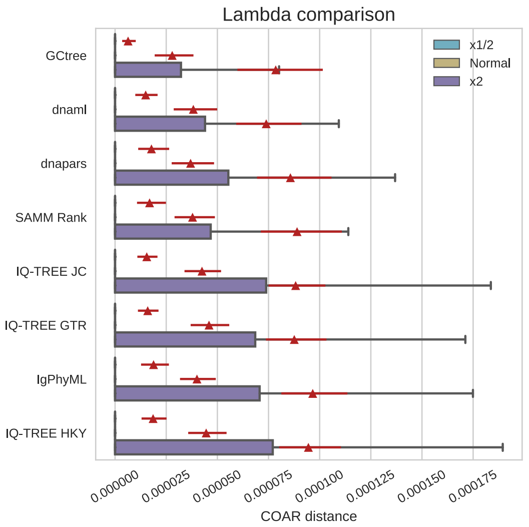

Figure 11: Neutral simulation showing COAR metric. Mutations were drawn from a uniform distribution over sites and substitutions using mutation rates: "x1/2" = 0.1825, "Normal" = 0.365, and "x2" = 0.73.

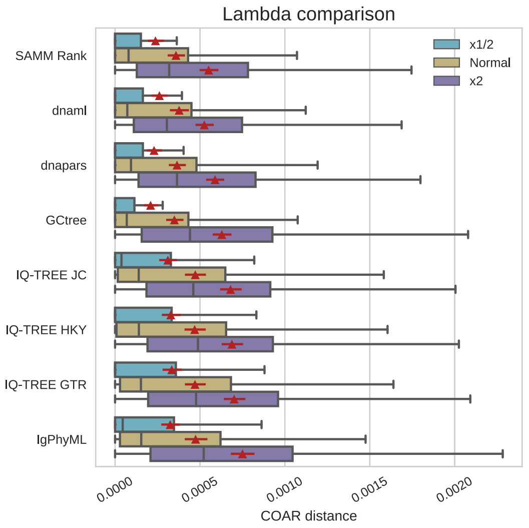

Figure 12: Affinity simulation showing COAR metric. Mutations were drawn from a uniform distribution over sites and substitutions using mutation rates: "x1/2" = 0.1825, "Normal" = 0.365, and "x2" = 0.73.

Comparing joint and marginal ancestral sequence reconstruction

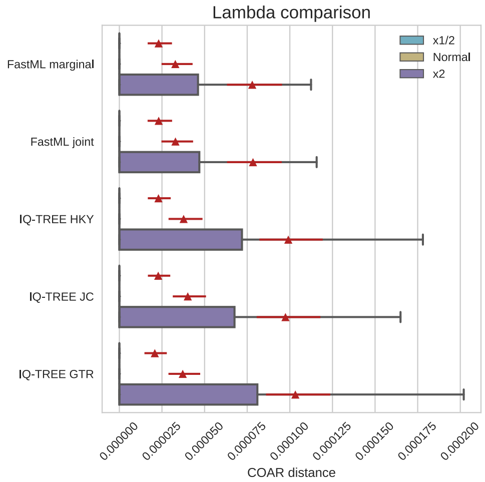

Figure 13: Comparing joint and marginal sequence reconstruction using FastML's neighbor joining method, with IQ-TREE as reference. Neutral simulation showing COAR metric for mutation rates: "x1/2" = 0.1825, "Normal" = 0.365, and "x2" = 0.73.

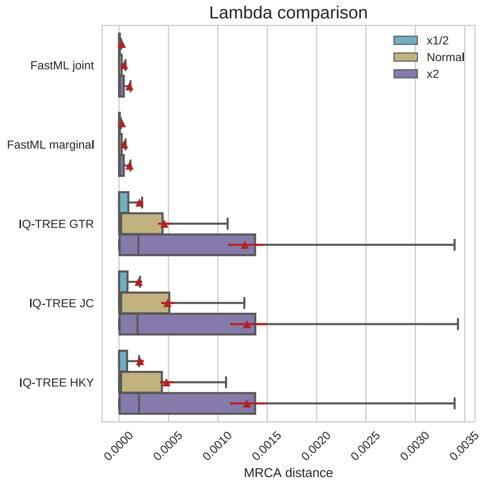

Figure 14: Comparing joint and marginal sequence reconstruction using FastML's neighbor joining method, with IQ-TREE as reference. Neutral simulation showing MRCA metric for mutation rates: "x1/2" = 0.1825, "Normal" = 0.365, and "x2" = 0.73.

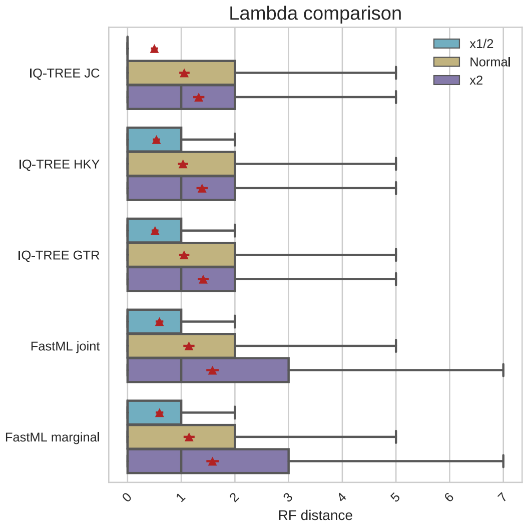

Figure 15: Comparing joint and marginal sequence reconstruction using FastML's neighbor joining method, with IQ-TREE as reference. Neutral simulation showing RF metric for mutation rates: "x1/2" = 0.1825, "Normal" = 0.365, and "x2" = 0.73.

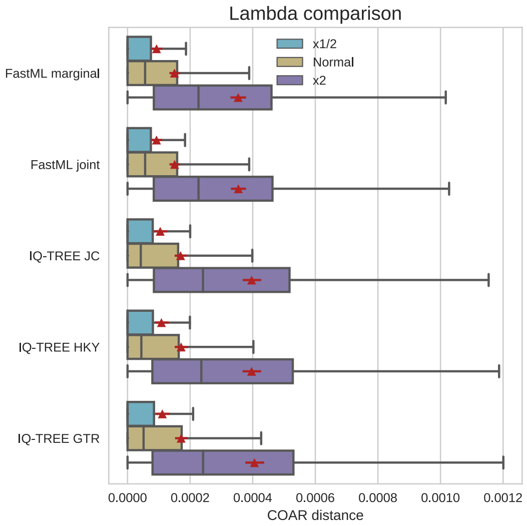

Figure 16: Comparing joint and marginal sequence reconstruction using FastML's neighbor joining method, with IQ-TREE as reference. Affinity simulation showing COAR metric for mutation rates: "x1/2" = 0.1825, "Normal" = 0.365, and "x2" = 0.73.

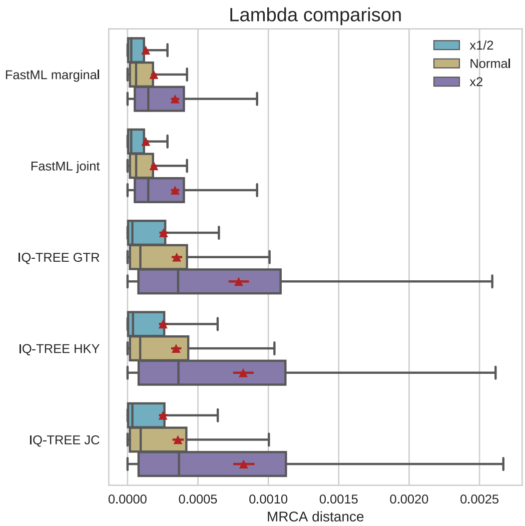

Figure 17: Comparing joint and marginal sequence reconstruction using FastML's neighbor joining method, with IQ-TREE as reference. Affinity simulation showing MRCA metric for mutation rates: "x1/2" = 0.1825, "Normal" = 0.365, and "x2" = 0.73.

Isotype score calculation

Method	dnaml	GCtree
Misplacement count	1	0
Baseline	0.672	0.691
Normalized score	1.488	0

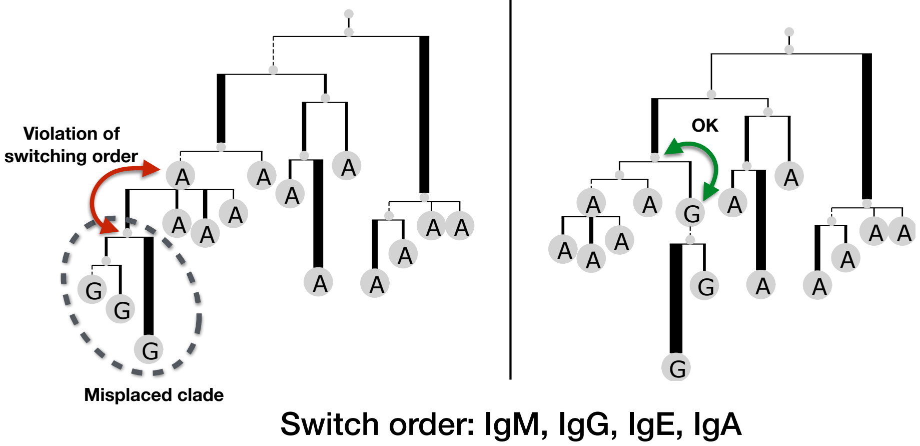

Figure 18: Example calculation of the isotype score. On the left: a tree inferred by dnaml where one clade has been misplaced resulting in a violation in the isotype switching order. One the right: a tree inferred by GCtree, on the same sequences, does not have any violations in the isotype switching order. The misplacement count is normalized by dividing it by a baseline score, found by taking the average misplacement score of 10,000 label-shuffled trees of the same topology. The normalized score is also referred to as the “isotype score”.

Isotype score comparison

The isotype score distribution was computed over 697 selected clonal families. The isotype score has a very high variance as can be observed in the 95% bootstrap confidence interval (10,000 replicates of sampling with replacement) of the mean. The comparison was run twice: once using the S5F motif model for SAMM ranking and another using SAMM's own 5-mer motif model fitted on the mutations in the 697 selected clonal families. For all other tools these represent replicate runs. The replicated runs clearly exemplifies the uncertainty of the mean estimates e.g. IQ-TREE under the JC model was ranked 4th in the S5F replicate and 7th in the SAMM replicate (Figure 19, upper row). The only consistent features are that IgPhyML ranks high (second best) and dnapars ranks lowest, however the differences between the non-dnapars tools seem hard to discern (Figure 19, lower row).

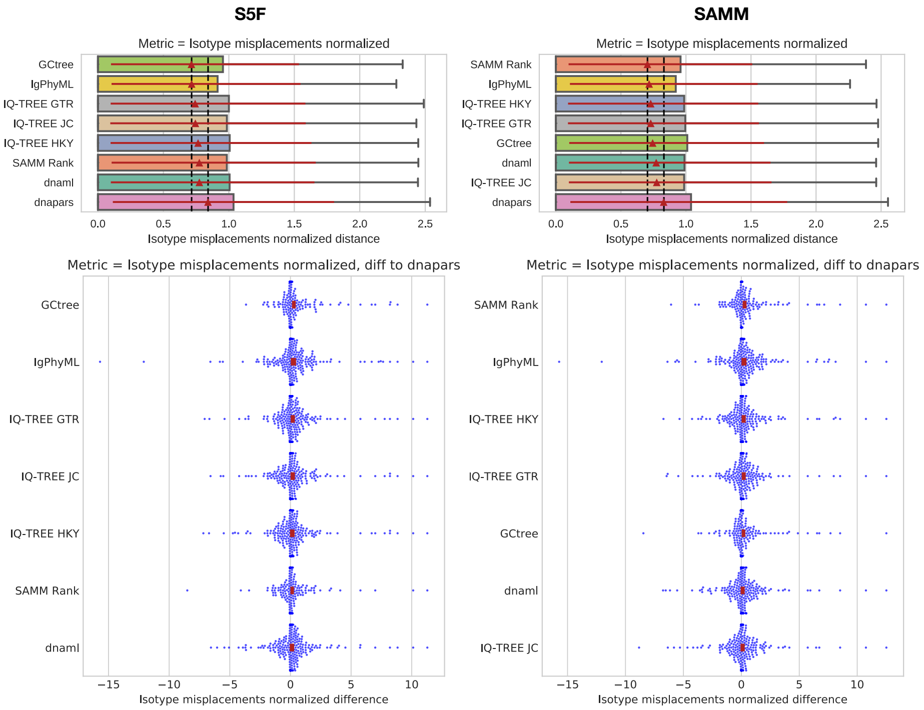

Figure 19: Isotype score distribution on 697 selected clonal families. In the left column: SAMM ranking uses the S5F model. In the right column: SAMM ranking uses its own motif model fitted on the mutations in the input data. Upper row shows the isotype score distribution, lower row shows the isotype score distributions of the non-zero isotype score differences between dnapars and all tools (positive means better than than dnapars).

Simulating affinity maturation

In this section we will describe our BCR sequence simulation framework in depth, first by introducing the neutral process which is the foundation of all our simulations, and then going on to motivate and derive a model that incorporates BCR affinity and antigen competition to define sequence fitness.

Neutral model

The neutral process can be viewed as a model of cell divisions, where at each cycle through the GC a cell can either die or produce a number of offspring, and each offspring has some probability of carrying mutations. Offspring numbers larger than two are used to approximate multiple cell divisions in a single GC cycle. The root sequence (naive BCR) is given at the simulation initialization as a starting point from where the tree is evolved until the simulation is stopped. Cell division is controlled by a Pois(λ) progeny distribution, and at each GC cycle all progeny cells will undergo a mutation process. The number of nucleotides to mutate is drawn from another Poisson distribution (Pois(λ_{mut})) and introduced sequentially into the sequence using a substitution model. Sequential introduction of mutations allows the possibility of back mutations. We use the S5F mutation model (1) to introduce mutations, which describes mutability and substitution preferences of the middle base of all 5-mer DNA motifs. However, a 5-mer mutability cannot be used directly on sites at the start or end of a sequence because of missing context, therefore we fill in missing context with the unknown base, N, and average over all possible motifs fitting into this ambiguous context.

Termination of the neutral branching process is achieved in either of three ways: 1) by simulating under a subcritical process ($\lambda < 1$) (2) and following it until extinction, 2) by using a stopping time T, or 3) by stopping after a population of N cells has been reached. Sequences are then sampled from the tree leaves. In addition we introduced a parameter for down-sampling the cell population to n cells. Model parameters are tabulated in Table 1.

Parameter	Description
λ	Pois(λ) progeny distribution
λ_{mut}	Pois(λ_{mut}) mutation distribution
T	Stopping time
N	Stopping number of sequences
n	Down-sampled number of sequences

Table 1: Parameters used in the neutral simulation.

Simulations with affinity selection

To model the affinity maturation process with selection we will use the exact same framework as described for the neutral process, but now the Pois(λ) progeny distribution is no longer constant. We consider the magnitude of λ as the fitness of a cell. In the neutral model λ is a fixed constant resulting in a completely flat fitness landscape, as opposed to a system with selection where

the fitness landscape is a more complex and rugged surface. The following subsections will describe a model with the simple purpose of defining a function to calculate the fitness of any BCR sequence. The fitness is measured in terms of a single $\lambda^{(i)}$ associated to each cell and defining a cell specific progeny distribution. Thus, selection is condensed into a dynamic λ, and this is the only difference to the neutral process.

Model concept and biological assumptions

Let us make some basic assumptions to keep later definitions simpler. First, the system we intend to model is the affinity maturation process happening in the GC, assumed to be driven by the BCR's affinity towards a single target antigen. A real GC reaction is seeded by 50-200 naive B cells, however, due to the extensive competition they often completely "resolve" in later stages of affinity maturation, resulting in cells with only a single common naive B cell ancestor i.e. monoclonallity (3). We do not attempt to model this inter-clonal competition so we make the simplifying assumption that the simulated GC is seeded by a single naive B cell. In our model it is the BCR amino acid sequence that is under selection, thus we ignore the possible fitness effects of synonymous mutations.

The GC is modeled with constant volume and constant total concentration of antigen. B cells compete for this limited antigen. B cells with high affinity BCRs will bind more antigen and are more likely to undergo cell division and vice versa for low affinity BCRs. Binding equilibrium is assumed to be instantaneous and the progeny distribution for a B cell is evaluated as a function of the BCR occupancy at this equilibrium. Affinity is a function of the BCR sequence and its amino acid sequence distance from the best BCR (here called the mature sequence). Once a new cell has been created this changes the binding equilibrium which then needs to be updated. A GC cycle in the simulation is defined by one iteration through all the cells to evaluates their progeny distributions. Cartoon overview in Figure 20.

Kinetic model of BCRs binding antigen

In the following we derive the fraction of a B cell's BCRs bound to the antigen in a GC (BCR occupancy). This is then extended to a situation with multiple B cells with different BCR affinities.

First, consider the BCRs of a single B cell as free molecules with a total concentration of $[B_{\text{total}}]$, then the BCR occupancy at equilibrium is:

$$B_{\text{bound}} = \frac{[AB]}{[B_{\text{total}}]}$$

Where $[AB]$ is the concentration of BCRs bound to antigen. We need to derive a solution to calculate B_{bound}.
The binding equilibrium between free antigen ($[A]$), free BCRs ($[B]$) and BCR bound antigen ($[AB]$) is:

$$[\mathrm{A}] + [\mathrm{B}] \underset{\mathrm{k_{off}}}{\overset{\mathrm{k_{on}}}{\rightleftharpoons}} [\mathrm{AB}] \tag{1}$$

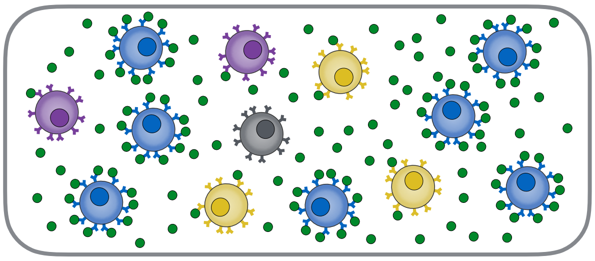

Name	Sequence	Distance
Mature	GGGVVQPGRSLRLSCAASGFTFSSYGMHWVRQAPGKGLEWVAVIWYDGSNKYYADSVKGRFTISRDNSKNTLYLQMNSLRAEDTAVYYCAGGGSYDGDYW	
	GGGVVQPGRSLRLSCAASGFTFSSYGMHWVRQAPGKGLEWVAVIWYDGSNKYYADSVKGRFTISRDNSKNTLYLQMNSLRAEDTAVYYCAGGGSYDGDYW	0
	GGGVVQPG**R**SLRLSCAASGFTFSSYGMHWVRQAPGKGLEWVAVIWYDGSNKYYADSVKGRFT**I**SRDNSKNTLYLQMNSLRAEDTAVYYCAGGGSYDGDYW	2
	GGGVVQPG**R**SLRLSCAASGFTFSSYGMHWVRQAPGK**G**LEWVAVIWYDGSN**K**YYADSVKGRFT**I**SRDNSKNTLYLQMNSLRAEDTAVYYC**A**GGGSYDGDYW	5
	GGGVVQPG**R**SLRL**S**CAASGFTFSSYGMHWVRQAPGK**G**LEWVAVIWYDGSN**K**YYADSVKGRFT**I**SRDNSK**N**TLYLQMNSLRAEDTAVYYC**A**GGGSYDGDYW	7

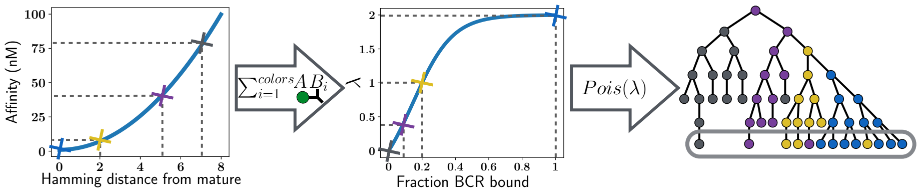

Figure 20: Simulation overview. The system is considered as a closed environment with free floating antigen and a number of B cells presenting BCRs on their surface, (top panel). Different colors correspond to different affinity BCR sequences. In the middle panel a sequence alignment shows the distance between BCR sequences and the mature BCR. Bottom panel shows first how distance from the mature BCR is converted to affinity, then how the fraction of bound BCRs is transformed to a λ defining the progeny distribution. Rightmost of the bottom panel shows the lineage tree with an ellipse marking the B cells of the current generation also displayed in the top panel.

The on- and off-rate of binding is expressed as constants k_{on} and k_{off}. Affinity can then be expressed as:

$$K_d \equiv \frac{k_{\text{off}}}{k_{\text{on}}} = \frac{[A][B]}{[AB]} \tag{2}$$

Isolating $[AB]$:

$$[AB] = [B]\frac{[A]}{K_d}$$

Substituting $[B]$ for its expression from mass conservation, $[B_{\text{total}}] = [B] + [AB]$:

$$[AB] = ([B_{\text{total}}] - [AB])\frac{[A]}{K_d}$$

Which rearranges to the result:

$$[AB] = \frac{[B_{\text{total}}]}{1 + \frac{K_d}{[A]}}$$

Then extending the model for binding equilibrium of a single BCR sequence to

one with multiple BCR sequences just requires indexing:

$$
\begin{aligned}
[\mathrm{A}] + [\mathrm{B}^{(1)}] &\underset{\mathrm{k}_{\mathrm{off}}^{(1)}}{\overset{\mathrm{k}_{\mathrm{on}}^{(1)}}{\rightleftharpoons}} [\mathrm{AB}^{(1)}] \\
[\mathrm{A}] + [\mathrm{B}^{(2)}] &\underset{\mathrm{k}_{\mathrm{off}}^{(2)}}{\overset{\mathrm{k}_{\mathrm{on}}^{(2)}}{\rightleftharpoons}} [\mathrm{AB}^{(2)}] \\
&\vdots \\
[\mathrm{A}] + [\mathrm{B}^{(n)}] &\underset{\mathrm{k}_{\mathrm{off}}^{(n)}}{\overset{\mathrm{k}_{\mathrm{on}}^{(n)}}{\rightleftharpoons}} [\mathrm{AB}^{(n)}]
\end{aligned}
$$

The same solution applied and because all B cells compete for the same antigen, each $[AB^{(i)}]$ is dependent through the concentration of unbound antigen:

$$
\begin{aligned}
[AB^{(1)}] &= \frac{[B_{\text{total}}^{(1)}]}{1+\frac{K_d^{(1)}}{[A]}} \\
[AB^{(2)}] &= \frac{[B_{\text{total}}^{(2)}]}{1+\frac{K_d^{(2)}}{[A]}} \\
&\vdots \\
[AB^{(n)}] &= \frac{[B_{\text{total}}^{(n)}]}{1+\frac{K_d^{(n)}}{[A]}}
\end{aligned} \tag{3}
$$

Now introducing mass conservation for the antigen A:

$$
A_{\text{total}} = [A] + \sum_{i=1}^{n} [AB^{(i)}] \equiv [A] + \sum_{i=1}^{n} \frac{[B_{\text{total}}^{(i)}]}{1 + \frac{K_d^{(i)}}{[A]}} \tag{4}
$$

By rearranging to a polynomial form the system can be solved by root finding to calculate $[A]$ which is then used to find all the $[AB^{(i)}]$'s and transformed them to $B_{\text{bound}}^{(i)}$'s.

This is a solution to a model of BCR competition in the GC but to make this work we also need a definition of BCR affinity as well as a way of transforming BCR occupancy to fitness in the sequence simulation.

Defining affinity for a sequence

Here we describe how to define the affinity ($K_d^{(i)}$) of each BCR. A numerical affinity value can be generated by transforming a BCR sequence ($S^{(i)}$) into a number that represents affinity. Formally, this would be a function: $f(S^{(i)}) = K_d^{(i)}$. Consider that the BCRs in a GC are evolving towards a specific mature sequence, denoted S^M. A mature sequence is the sequence with the highest affinity and fitness. We will define a fitness landscape around this mature sequence using Hamming distance between amino acid sequences of the mature and the evaluated sequence: $d_H(\cdot, \cdot)$. We take it as a prerequisite that the evaluated sequence is functional i.e. contains no stop codon, and therefore

should the mutation process return a nonsense mutated sequence it gets assigned zero affinity. In conditions with limiting antigen this acts as a strong selection against nonsense mutated sequences.

Let us define the affinity of the naive input sequence as K_d^N and correspondingly the affinity for the mature sequence as K_d^M. Now, we can define an arbitrary function with reference points in K_d^N and K_d^M, that transforms a distance between $S^{(i)}$ and S^M to an affinity:

$$f(S^{(i)}, d_0, S^M, K_d^N, K_d^M) = K_d^{(i)}$$

Where $d_0 = d_H(S^N, S^M)$ is the distance between the naive and mature sequences. There are two constraints we want to impose. If the BCR sequence is: 1) equal to the naive sequence (S^N) it takes the affinity of the naive BCR (K_d^N), and 2) equal to the mature sequence (S^M) it takes the affinity of the mature BCR (K_d^M):

$$\begin{aligned} f(S^N, d_0, S^M, K_d^N, K_d^M) &= K_d^N \\ f(S^M, d_0, S^M, K_d^N, K_d^M) &= K_d^M \end{aligned} \tag{5}$$

A flexible function for transforming distance to affinity is the family of power transformations which we define with the two conditions satisfied as:

$$f(S^{(i)}, d_0, S^M, K_d^N, K_d^M) = K_d^M + \left(\frac{d}{d_0}\right)^k (K_d^N - K_d^M) \tag{6}$$

Where $d = d_H(S^{(i)}, S^M)$ is the distance between the input and mature sequences. The exponent, k, can be chosen to adjust the mapping between distance and affinity, with the restriction that $0 < k < \infty$ (Figure 21).

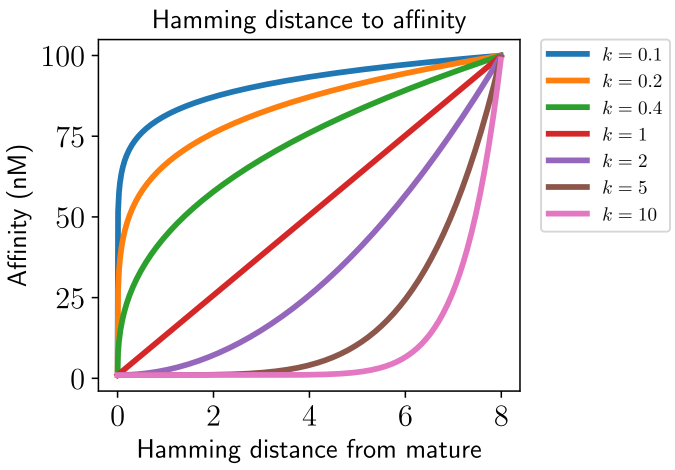

Figure 21: Varying the exponent k in (6) to achieve different mappings between distance and affinity. Naive and mature affinity is held constant, $K_d^N = 100nM$ and $K_d^M = 1nM$.

In a real affinity maturation process there may be many different BCR sequences that are practically equally fit e.g. this will happen when multiple amino acids are equally fit on a given position, and it will also happen if there are multiple distinct maturation paths that end up with equally fit BCRs. Our model deals with this by allowing multiple mature sequences and then determining the affinity based on the shortest distance to any of these:

$$d = \underset{S^M \in \text{mature sequences}}{\text{argmin}} d_H(S^{(i)}, S^M)$$

Transforming BCR occupancy to fitness

Equipped with a sequence to affinity mapping and a method to solve the binding equilibrium in a population of BCRs the last element necessary is to couple BCR occupancy to fitness. This is achieved through the progeny distribution; if $B^{(i)}_{\text{bound}}$ is small the progeny distribution should favor terminating the B cell and opposite, if $B^{(i)}_{\text{bound}}$ is large the progeny distribution should favor cell division. The Poisson distribution will reflect this behavior by setting $\lambda^{(i)}$ small when $B^{(i)}_{\text{bound}}$ is small and $\lambda^{(i)}$ large when $B^{(i)}_{\text{bound}}$ is large. However, it is unrealistic that there should be a one-to-one mapping between $B^{(i)}_{\text{bound}}$ and $\lambda^{(i)}$ and therefore we need a function for transformation: $Y(B^{(i)}_{\text{bound}}) = \lambda^{(i)}$. The function should allow specification of lower and upper bounds on $\lambda^{(i)}$, a threshold (f_{full}) on $B^{(i)}_{\text{bound}}$ when more bound antigen does not have any fitness effects (Figure 22) and another threshold ($\frac{f_{\text{full}}}{U}$) defining $B^{(i)}_{\text{bound}}$ when the progeny distribution transitions between a subcritical and a supercritical process ($\lambda^{(i)} = 1$) (2) (Figure 23). These requirements can be accommodated by the generalized logistic function:

$$\lambda^{(i)} = Y(B^{(i)}_{\text{bound}}) = \alpha + \frac{K - \alpha}{G + Q\exp(-\beta B^{(i)}_{\text{bound}})} \tag{7}$$

G is chosen to be the typical logistic function value of 1. K is the upper bound on $\lambda^{(i)}$ and is set to 2 (slightly larger than the $\lambda = 1.5$ fitted for the neutral branching process). α, β and Q are found using three conditions:

$$Y(0) = 0, \quad Y\left(\frac{f_{\text{full}}}{U}\right) = 1, \quad Y(f_{\text{full}}) = 2 - \epsilon \tag{8}$$

The solution is undefined in $Y(f_{\text{full}}) = 2$ because the function is asymptotically growing towards 2, therefore ϵ can be regarded as a small value (e.g. 10^{-3}) so that $Y(f_{\text{full}}) \approx 2$. The constant U in condition 2 can be adjusted to set the value of $B^{(i)}_{\text{bound}}$ resulting in $\lambda^{(i)} = 1$. Using these conditions α, β and Q can be found and the logistic function is fully defined. α can be interpreted as the lower asymptote of the function. β is the steepness of the function and it is coupled to the Q parameter and follows it according to the three conditions in (8).

Parameter choices

We define the maximum fitness to be attained at 100% BCR binding, hence we fix $f_{\text{full}} = 1$. The infliction point parameter U is chosen to reflect our expectation

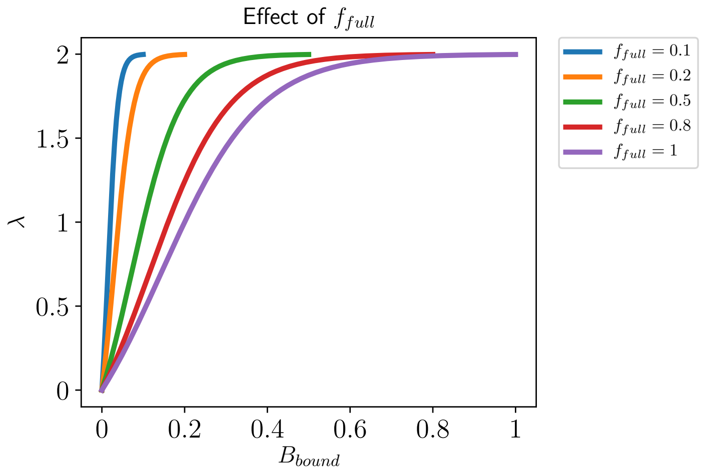

Figure 22: Using a constant $U = 5$, changing the f_{full} parameter in the conditions in (8) to change the point where B_{bound} reaches the λ plateau.

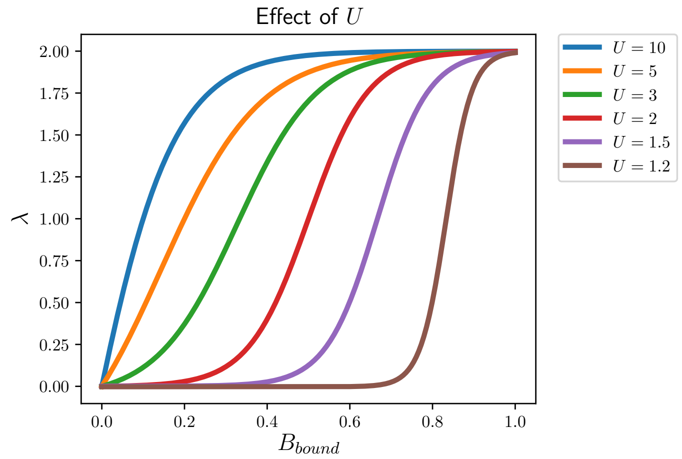

Figure 23: Using a constant $f_{\text{full}} = 1$, changing the U parameter in the conditions in (8) to achieve a shift of the inflection point at $\lambda = 1$ on the B_{bound} axis.

that initially, when only a few BCRs are bound and stimulation is low, there will be a linear increase of the stimulus when antigen binding increase, and at some point close to f_{full} the increase in stimulus levels out. This expected shape is recapitulated by a choosing $U = 5$ (Figure 23).

The total concentration of antigen (A_{total}) needs to be defined to solve the binding equilibrium. To do this we need to introduce the concept of a carrying capacity of the simulated GC, which is defined as the number cells a GC is able to support in its micro environment. The carrying capacity is determined mainly by the total concentration of antigen since binding to antigen controls

the progeny distribution. BCR affinity is also influencing antigen binding and therefore must influence the carrying capacity, but at high affinity nearly all antigens are bound and hence the total antigen concentration is the most influential determinant of GC carrying capacity. At Pois(1) the progeny distribution is only just sustaining the population size of the GC, and given condition 2 in (8) this happens at $\frac{f_{\text{full}}}{U}$. Then, under the assumption that the population of B cells all have identical BCR sequences, the maximum carrying capacity is:

$$C([A_{\text{total}}]) = \frac{U}{f_{\text{full}}} \frac{A_{\text{total}} - [A]}{B_{\text{total}}} \approx \frac{U}{f_{\text{full}}} \frac{A_{\text{total}}}{B_{\text{total}}} \tag{9}$$

Using a carrying capacity of 1000 (4, 5) we can calculate A_{total}. We note that simulations are generally robust to different parameter choices (Figure 24).

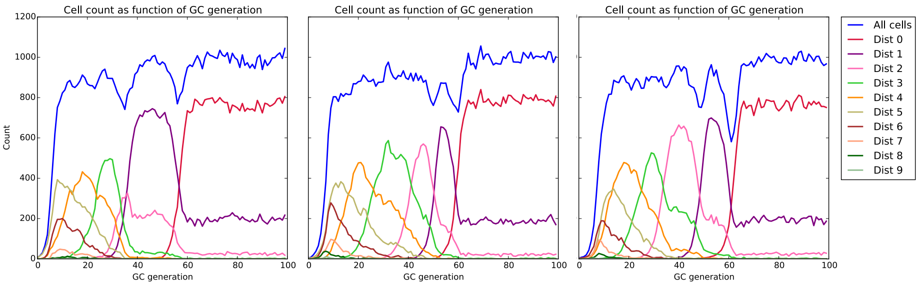

(a) (b) (c)

Figure 24: Simulation with affinity selection for varying magnitudes of f_{full}. (a) $f_{\text{full}} = 1$, (b) $f_{\text{full}} = 0.5$ and (c) $f_{\text{full}} = 0.05$. Simulations with $d_0 = 10$, $U = 5$ and $[A_{\text{total}}]$ adjusted to obtain a carrying capacity of 1000 cells. Each simulation was run for 100 generations and the composition of sequence distances to their closest mature sequence are plotted for each generation.

In the transformation from distance to affinity in (6), we have to make a choice about which exponent to use. We would like to disallow sequences drifting far away from the mature sequence by enforcing a positive exponent. Furthermore, we require that each Hamming distance step between the naive and mature sequences has a substantial affinity effect, and therefore $k = 2$ is used.

The amino acid sequence distance between the naive and mature sequences, d_0, is set to 5. The K_d for a naive sequence is likely in the low micro molar range range of $10^{-6} - 10^{-7}M$, while the mature affinity is in the nano or subnano molar range of $10^{-8} - 10^{-10}M$ (6–9) (M is used to denote molar concentration). We choose the naive sequence to be $K_d^{\text{naive}} = 10^{-7}M$ $(100nM)$ and the mature to be $K_d^{\text{mature}} = 10^{-9}M$ $(1nM)$, giving a large span in affinity to select on. Based on approximating the GC as spheric, and using the experimental data for average GC diameter and BCRs per B cell, the model is fully defined in nanomolar concentrations. All necessary constants are tabulated in Table 2.

Constant	Value	Description	Reference
B_{total}	1×10^4	Number of BCRs on each B cell	(10, 11)
n_t	1000	B cells per GC	(4, 5)
dim	$10^{-4}m$	GC diameter	(12)
$\frac{1}{U}$	$\frac{1}{5}$	Fraction of f_{full} necessary to sustain the population	See text
d_0	5	Distance between the naive and mature sequences	See text
k	2	Exponent of affinity transformation	See text
f_{full}	1	Fraction BCRs bound at full activation	See text
K_d^{naive}	100nM	Naive affinity	(6–9)
K_d^{mature}	1nM	Mature affinity	(6–9)

Table 2: Constants used in the model of affinity simulation.

Simulation summary statistics

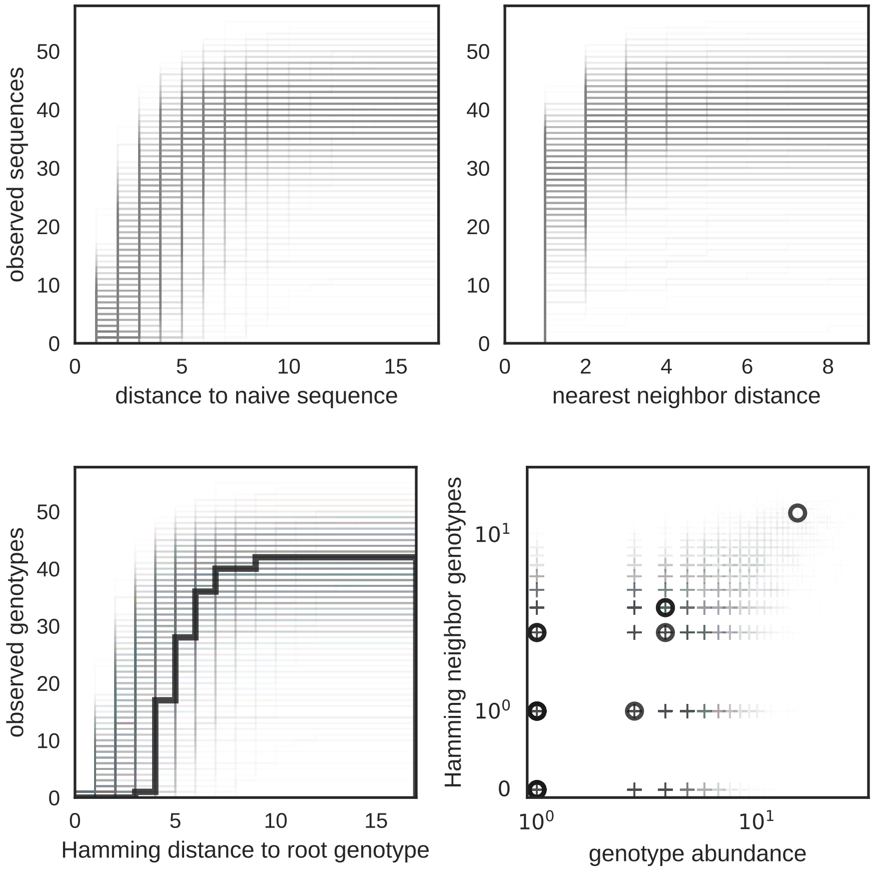

Figure 25: Summary statistics for sequences simulated under the neutral model with the mutation rate parameter set to 0.365. In the upper row: cumulative density functions (CDFs) showing the distribution of sequences according to distance from the naive sequence and the nearest neighbor sequence. In the lower row: distance to root CDFs and a scatter plot showing the correlation between genotype abundance and the number of single edit distance neighbors ("Hamming neighbor"). In black bold is the statistics for the single germinal center sequence dataset from (3). Each line represents a simulation.

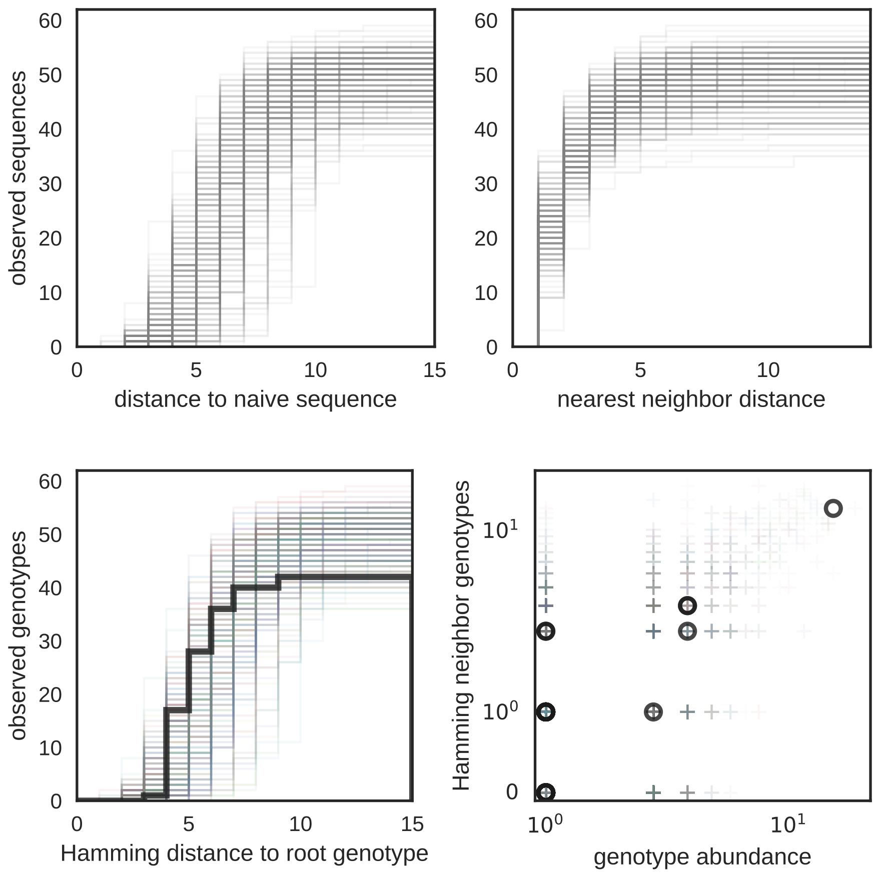

Figure 26: Summary statistics for sequences simulated under the affinity model with the mutation rate parameter set to 0.365. In the upper row: cumulative density functions (CDFs) showing the distribution of sequences according to distance from the naive sequence and the nearest neighbor sequence. In the lower row: distance to root CDFs and a scatter plot showing the correlation between genotype abundance and the number of single edit distance neighbors (“Hamming neighbor”). In black bold is the statistics for the single germinal center sequence dataset from (3). Each line represents a simulation.

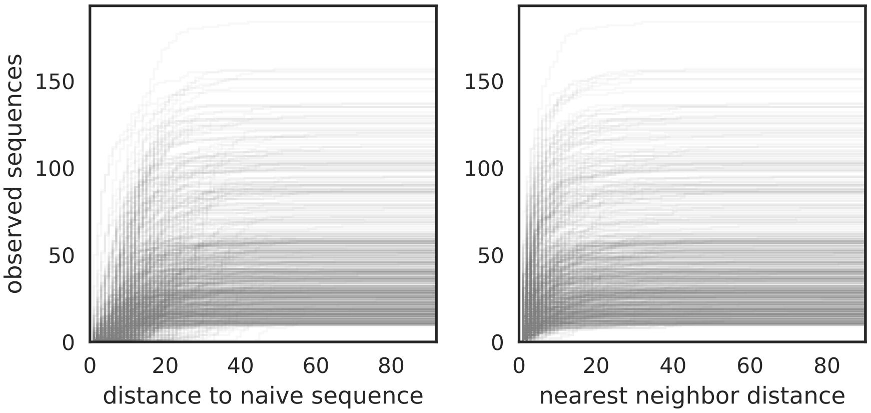

Figure 27: Summary statistics for sequences in the isotype dataset. Cumulative density functions (CDFs) showing the distribution of sequences according to distance from the inferred naive sequence and the nearest neighbor sequence. Each line represents a clonal family.

Correctness of ancestral reconstruction

In the following section we will introduce a benchmark metric for ancestral sequence reconstruction, which we call “correctness of ancestral reconstruction” (COAR). The correctness of a reconstruction compared to the true evolutionary history can be measured by multiple similarity measures e.g. topological similarity, branch length similarity and sequence similarity between inferred and real ancestors. All these measures are inter-dependent e.g. the inferred sequences are affected by the branch lengths and the topology and the branch lengths are conditioned on a topology etc. And while inferring correct tree topology is important in its own right, the correctness of the inferred ancestral sequences are the foremost important objective of most BCR phylogenies when these sequences are used for applications involving DNA synthesis, protein expression and functional testing. For this reason, the sole purpose of the COAR metric is to capture the correctness of the inferred ancestral sequences. In particular, we would like to propose a loss function that does not penalize a phylogeny when minor parts of the tree topology is incorrect while ancestral sequence reconstruction is perfect.

The purpose of COAR is to compare two trees built with the same leaves; let us call these the true and inferred tree. When performing ancestral sequence reconstruction the desired result is often to reconstruct the internal nodes in the direct path going from a leaf to the root, as illustrated in Figure 28. This path is extracted by starting at a leaf node and traversing upwards, parent by parent, until the root is reached. In the following, this list of sequences will be referred to as the ancestral lineage. The correct ancestral lineage is the objective of COAR, and we construct the COAR value so it represents the expected per-site error in such a reconstruction. Following the example in Figure 28, often there will be small differences in tree topology between the true and inferred trees, and these will likely make the number of internal states in the ancestral lineages differ. This makes comparison difficult because two lists of different length cannot be element-wise compared. The lists could be made equal length by adding gaps, but then a systematic way of adding these would be necessary.

The basis of COAR is a list comparison progressing element-wise through the list i.e. element 1 in list 1 compared to element 1 in list 2, next, element 2 in list 1 compared to element 2 in list 2 etc. For lists of similar length the list comparison is easy, it will simply be the cumulated distance from list element comparisons, corresponding to the sum of Hamming distances between inferred and true ancestors in the lists. When lists are not equally long, one or more gaps must be introduced into one of the lists; we choose to do so in such a way that the list similarity is maximized. This is an alignment problem with matches/mismatches/gaps and it can be efficiently solved using the Needleman-Wunsch algorithm (13). We define it as a global alignment so that it has to start at the root and end at the leaf because both states are known for the true and inferred phylogenies. We further restrict the Needleman-Wunsch algorithm so that gaps are only allowed to be introduced into the shortest of the two lists being aligned, this forces the maximum number of node comparisons.

One interpretation of the COAR value is that it is the distance between the true and inferred mutation histories, as illustrated in in Figure 29. In this representation of an ancestral lineage the root and the leaf are two fixed states with a continuous mutation process running between them. The internal nodes

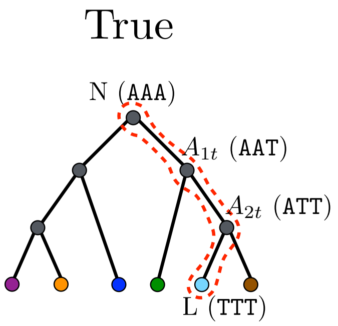

Figure 28: True vs. inferred tree with colored leaves and grey ancestral states. Reconstruction from the light blue leaf is marked by a dashed red line and annotated with genotypes in parenthesis. N is the naive sequence, L is the leaf sequence and the As are ancestors $1, 2, \ldots, n$ with either true or inferred marked by t or i, respectively, appended to the subscript. The inferred tree has misplaced the branch leading to the light blue node, resulting in a missing ancestral sequence.

in the ancestral lineage are discrete states in the continuous process, on the true tree these corresponds to actual cells but on the inferred tree they need not correspond to actual observed genotypes. Instead we can think about them as realizations along the continuous mutation process defined by the inferred tree. The COAR value is then a similarity measured between the true cell genotype and the inferred realizations, each sampled from the true and inferred mutation processes respectively, and in the case of a mismatch between the number of realizations and cells, a gap will be introduced in the alignment to compensate.

Using the aligned ancestral lineages it is now possible to derive a score, similar to a sequence alignment score. We use negative penalties for mismatches and zero points for matches, and furthermore normalize the alignment score to the smallest possible score (all mismatches) for that lineage, yielding the COAR value for a single lineage i:

$$\text{COAR}_i = \frac{\text{alignscore}(\text{leaf}_i)}{\text{alignscore}_{\text{min}}(\text{leaf}_i)}$$

Where alignscore is the score of the alignment between the true and inferred ancestral lineages and alignscore_{min} is the smallest possible score given the number and length of the sequences in the ancestral lineages. The alignment score is defined in terms of penalties, so all values are less than or equal to zero. Since both numerator and denominator are negative the COAR value is positive.

COAR is defined in the range from 0 to 1, where 0 is a perfect ancestral sequence reconstruction and 1 is the worst. The COAR value is comparable across different trees, methods and datasets because of this normalization. Its value can be interpreted as the average per-site error across all the inferred ancestral lineage sequences. COAR for a single ancestral lineage can be expanded to the tree level by calculating the mean COAR value for the whole tree:

$$\text{mean}(\text{COAR}) = \sum_{i=1}^{N_L} \frac{\text{alignscore}(\text{leaf}_i)}{\text{alignscore}_{\text{min}}(\text{leaf}_i)} \Bigg/ N_L$$

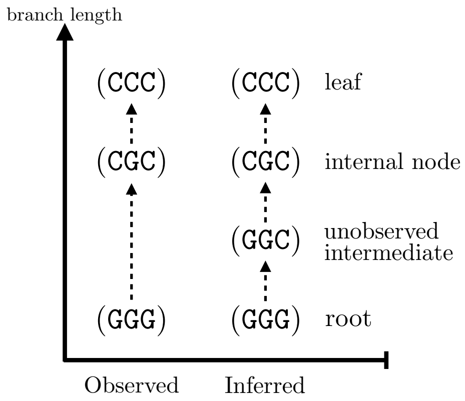

Figure 29: One interpretation of the COAR value is that it is the distance between the true and inferred mutation histories, here shown by the true and inferred ancestral lineage nodes of an example phylogeny. The true ancestral lineage (left side) represents actual observed cells where the genotype is a known constant. The inferred ancestral lineage (right side) represents the estimated genotypes at branching points along the inferred topology. In some cases there is a mis-correspondence between observed cells in the true phylogeny and the branching points in the inferred tree. These are treated as missing realizations and ignored in the alignment of the two mutation histories.

Where N_L is the number of leaves on the tree.

Calculating COAR values - example with a known root

As an example of how the COAR metric works we will present a small example, summarized in Figure 28 with the light blue leaf chosen for lineage reconstruction and the true and inferred ancestral lineages marked in each tree with red dashed lines. The root sequence is a known state called the naive sequence. Assume that the true phylogeny is known with corresponding ancestral sequences. Now take a leaf sequence on the tree and reconstruct its ancestral lineage by extracting the parent, the parent's parent, etc. until the root is reached, tabulated in Table 3. This ordered list of sequences constitute the reconstructed ancestral lineage for the chosen leaf and it always starts at the root and ends at the leaf, therefore we are imposing this as a restriction on the alignment. Furthermore, these two known states they do not count towards the COAR value.

	True	Inferred
Naive (N)	AAA	AAA
A_1	AAT	TAT
A_2	ATT	-
Leaf (L)	TTT	TTT

Table 3: Reconstructed ancestral lineage for true and inferred trees as shown and marked by red dashed line in Figure 28.

In the case of a wrongly inferred topology the true and inferred list of ances-

tral lineage sequences can have different length. It is therefore necessary to find a way of getting the best possible alignment between these two lists. We know the start and end of this alignment but the sequences in between are free to be shifted up or down to maximize the alignment fit. We adapt the Needleman and Wunsch dynamic program solution (13) to solve this as an alignment problem. A notable difference to the original algorithm is that it was intended to align two sequences of characters, like DNA or amino acids, while in this application a list of whole sequences are aligned.

The first step in the alignment algorithm is to calculate a score matrix of all pairwise sequence comparisons. For this example we use the negative Hamming distance as a score, however, the score function can be extended to reflect different situations, like imposing a larger penalty for non-synonymous rather than synonymous mutations. The score matrix is tabulated in Table 4.

	N	A_{1t}	A_{2t}	L
N	0	-1	-2	-3
A_{1i}	-2	-1	-2	-1
L	-3	-2	-1	0

Table 4: Score matrix based on all pairwise distances between the sequence in Figure 28.

Now we are ready to initializing the alignment grid used in the dynamic programming solution of the alignment problem. Initialization is started by inserting the scores of pure gap characters i.e. first row and first column (Table 5), and we enforce alignment of the two root sequences by setting these gap penalties to negative infinity. Similarly, we disallow introduction of gaps in the longest of the two lists, also by penalizing with negative infinity (Table 6). Setting certain gap penalties to negative infinity is a simple way of dealing with disallowed gaps and it also works well for implementations.

	-	N	A_{1t}	A_{2t}	L
-	0	-Inf	-Inf	-Inf	-Inf
N	-Inf	$\multimap$			
A_{1i}	-Inf				
L	-Inf				

Table 5: The starting alignment grid, initialized with negative infinite gap penalties to disallow gap opening in the beginning of the alignment. The grid is filled up from left to right row by row, starting in the cell marked by $\multimap$.

Then the alignment grid is filled up, starting with the cell marked by $\multimap$ in Table 5, progressing to the rightmost cell and continuing in the same fashion on the next row. Cells are filled up using the following maximization:

$$C_{i,j} = \max\{(C_{i-1,j} + gp_{\text{down}}); (C_{i,j-1} + gp_{\text{right}}); (C_{i-1,j-1} + S_{i-1,j-1})\}$$

Where $C_{i,j}$ is the ith row and jth column cell in the grid, gp_{down} is the penalty of making a downwards gap, gp_{right} is the penalty of making a rightwards gap and $S_{i-1,j-1}$ is the score of aligning the ith, jth elements found by look-up in

the score matrix (Table 4) In this example the longest list is that of the true ancestral lineage so in this list gaps are disallowed. In the inferred lineage gaps are allowed but not penalized: $gp_{\text{down}} = -\text{Inf}$ and $gp_{\text{right}} = 0$.

The grid is filled and the final alignment score is the number in the rightmost bottom cell (Table 6).

	-	N	A_{1t}	A_{2t}	L
-	0	-Inf	-Inf	-Inf	-Inf
N	-Inf	0	0	0	0
A_{1i}	-Inf	-Inf	-1	-1	-1
L	-Inf	-Inf	-Inf	-2	-1

Table 6: The filled alignment grid, ready for tracing back the best alignment. The rightmost bottom cell contains the score for the best alignment.

The last step is to traceback the best path through the alignment grid and return this as the list alignment. The traceback starts from the leaf sequence, in the right bottom corner, and ends with the naive sequence in the left top corner. A diagonal step is equivalent to a sequence match, a left move is introducing a gap character in the inferred list and a move up is introducing a gap in the true list. The best path is found by progressively moving upwards, choosing the move with:

$$\text{move}_{i,j} = \text{which}\left\{C_{i,j} = [(C_{i-1,j} + gp_{\text{down}}), (C_{i,j-1} + gp_{\text{right}}), (C_{i-1,j-1} + S_{i-1,j-1})]\right\}$$

Notice that this path has already been generated when the alignment grid was filled up and could be cached. The resulting alignment and the penalty for each position is tabulated in Table 7.

Lastly the alignment score is normalized by the smallest possible alignment score i.e. no similarity between sequences in the lists. This normalized number is the COAR value and it runs between 0 to 1. In the presented example we only calculated the COAR value for the reconstructed ancestral lineage from one leaf, but by doing the calculations on all leaves on the tree and taking the average, the mean COAR value for the whole tree would be computed.

True<br>Inferred	N<br>N	A_{1t}<br>A_{1i}	A_{2t}<br>-	L<br>L
Penalty	0	-1	0	0
Max penalty	0	-3	0	0
COAR	-1/-3=0.333			

Table 7: The resulting alignment and the penalties for each position.

References

[1] Cui A, Di Niro R, Vander Heiden JA, Briggs AW, Adams K, Gilbert T, et al. A Model of Somatic Hypermutation Targeting in Mice Based on High-Throughput Ig Sequencing Data. The Journal of Immunology. 2016;197(9):3566–3574.

[2] Harris TE. The theory of branching processes. Courier Corporation; 2002.

[3] Tas JM, Mesin L, Pasqual G, Targ S, Jacobsen JT, Mano YM, et al. Visualizing antibody affinity maturation in germinal centers. Science. 2016;351(6277):1048–1054.

[4] Kroese F, Timens W, Nieuwenhuis P. Germinal center reaction and B lymphocytes: morphology and function. In: Reaction Patterns of the lymph node. Springer; 1990. p. 103–148.

[5] Childs LM, Baskerville EB, Cobey S. Trade-offs in antibody repertoires to complex antigens. Phil Trans R Soc B. 2015;370(1676):20140245.

[6] Berek C, Milstein C. Mutation drift and repertoire shift in the maturation of the immune response. Immunological reviews. 1987;96(1):23–41.

[7] Kuraoka M, Schmidt AG, Nojima T, Feng F, Watanabe A, Kitamura D, et al. Complex antigens drive permissive clonal selection in germinal centers. Immunity. 2016;44(3):542–552.

[8] Phan TG, Paus D, Chan TD, Turner ML, Nutt SL, Basten A, et al. High affinity germinal center B cells are actively selected into the plasma cell compartment. Journal of Experimental Medicine. 2006;203(11):2419–2424.

[9] Ulrich HD, Mundorff E, Santarsiero BD, Driggers EM, Stevens RC, Schultz PG. The interplay between binding energy and catalysis in the evolution of a catalytic antibody. Nature. 1997;389(6648):271–275.

[10] Rieckmann JC, Geiger R, Hornburg D, Wolf T, Kveler K, Jarrossay D, et al. Social network architecture of human immune cells unveiled by quantitative proteomics. Nature immunology. 2017;18(5):583.

[11] Rieckmann JC, Geiger R, Hornburg D, Wolf T, Kveler K, Jarrossay D, et al. Social network architecture of human immune cells unveiled by quantitative proteomics. Nature Immunology. 2017;.

[12] Romppanen T. A morphometrical method for analyzing germinal centers in the chicken spleen. APMIS. 1981;89(1-6):263–268.

[13] Needleman SB, Wunsch CD. A general method applicable to the search for similarities in the amino acid sequence of two proteins. Journal of molecular biology. 1970;48(3):443–453.
